# Untapped Mycobiota: A Scoping Review of Endophytic Fungi in Medicinal Plants from Malaysia

**DOI:** 10.3390/jof12070494

**Published:** 2026-07-05

**Authors:** Ling Yang, Chia Wei Phan, Yee Shin Tan, Jaya Seelan Sathiya Seelan

**Affiliations:** 1 Department of Pharmaceutical Life Sciences, Faculty of Pharmacy, Universiti Malaya, Kuala Lumpur 50603, Malaysia; lingyang986@gmail.com; 2 Mushroom Research Centre, Universiti Malaya, Kuala Lumpur 50603, Malaysia; 3 Institute of Biological Sciences, Faculty of Science, Universiti Malaya, Kuala Lumpur 50603, Malaysia; 4 Institute for Tropical Biology and Conservation, Universiti Malaysia Sabah, Jalan UMS, Kota Kinabalu, Sabah 88400, Malaysia; seelan80@ums.edu.my

**Keywords:** endophytic fungi, medicinal plants, Malaysia, taxonomic diversity, bioactivity screening, secondary metabolites

## Abstract

Endophytic fungi from Malaysian medicinal plants constitute a metabolically prolific yet underexplored reservoir for natural product discovery. This scoping review of 56 studies published between 2015 and 2025 identified a fundamental methodological divergence within the field: while phenotypic bioactivity screening dominates the literature (>87% of studies), it is weakly supported by chemical characterization (<25%) and entirely disconnected from genomic investigation (0% biosynthetic gene cluster studies). This phenotype-first paradigm has largely confined the field to descriptive reporting, limiting mechanistic understanding and translational potential. Collectively, the evidence reveals a substantial disconnect between reported bioactivities and their underlying biosynthetic foundations. To address this limitation, a practical genotype-to-phenotype workflow is proposed that integrates strain prioritisation, multi-omics-guided activation, chemical mapping, and mechanism-oriented validation. By linking genomic potential with metabolite production and biological function, this framework provides a roadmap for advancing fungal natural product discovery beyond conventional phenotype-driven screening. Adoption of such approaches may improve the identification of chemically novel and biologically relevant metabolites while supporting the sustainable development of Malaysia’s endophytic fungal resources for biotechnological and pharmaceutical applications.

## 1. Introduction

Endophytic fungi, defined as asymptomatic microorganisms residing within living plant tissues, are increasingly recognized as prolific producers of structurally diverse and biologically active secondary metabolites [1]. Medicinal plants represent a particularly compelling ecological context for these fungi, as long-term exposure to host-derived bioactive metabolites, environmental stress, and pathogen pressure is believed to selectively shape the composition and metabolic capability of their endophytic communities [2,3]. Consequently, endophytic fungi isolated from medicinal hosts frequently demonstrate antimicrobial, antioxidant, anticancer, anti-inflammatory, and enzyme-modulating activities, underscoring their potential as sustainable and complementary sources of bioactive compounds [4]. This promise is especially significant in biodiversity-rich tropical regions, where high plant endemism and ecological complexity are anticipated to harbour correspondingly diverse and underexplored fungal metabolomes [5]. Malaysia, a globally recognized biodiversity hotspot with a rich ethnobotanical heritage, provides an exceptional yet underexplored resource for the discovery of bioactive metabolites from endophytic fungi [6].

Over the past decade, increasing attention has been directed toward endophytic fungi associated with Malaysian medicinal plants, resulting in a growing body of literature reporting diverse fungal taxa and a wide range of biological activities. Despite this progress, the research landscape remains fragmented and methodologically uneven. Existing studies vary considerably in sampling design, taxonomic resolution, bioactivity evaluation, chemical characterization, and mechanistic investigation, making it difficult to determine whether current evidence collectively represents a coherent and mature body of knowledge. Importantly, the relationship between reported biological activities and their underlying biosynthetic capacity remains poorly understood. While antimicrobial, antioxidant, cytotoxic, and other bioactivities are frequently reported, the genomic basis of these phenotypes has received little attention within the Malaysian research context. In particular, the role of biosynthetic gene clusters (BGCs), which serve as the genetic foundation for fungal secondary metabolite production, remains largely unexplored [7]. This persistent disconnect between phenotype-based discovery and genotype-informed interpretation represents a major barrier to translational progress and limits the ability to prioritize promising strains for natural product development.

Given these challenges, a scoping review is needed to systematically map the current state of knowledge, evaluate methodological trends, identify structural research gaps, and establish future priorities for the field. Accordingly, this review examines studies published between 2015 and 2025 on endophytic fungi isolated from Malaysian medicinal plants. Specifically, it aims to (i) characterize the taxonomic diversity and host associations reported to date, (ii) summarize documented bioactivities and secondary metabolites, (iii) assess the extent of chemical and mechanistic elucidation, and (iv) evaluate how effectively current studies integrate taxonomic identification, bioactivity screening, chemical characterization, and genomic investigation.

## 2. Materials and Methods

### 2.1. Literature Search Strategy

This scoping review followed the Preferred Reporting Items for Systematic Reviews and Meta-Analyses extension for Scoping Reviews (PRISMA-ScR) guidelines [8]. A scoping methodology was chosen due to substantial heterogeneity across the available literature in experimental design, sampling approach, fungal identification, and bioactivity profiling, which precluded formal meta-analysis [9]. A comprehensive literature search was performed in February 2025 using three electronic databases: PubMed, Scopus, and Web of Science. These databases were selected to ensure broad coverage of biomedical, life sciences, and multidisciplinary research. The search strategy combined terms related to endophytic fungi, medicinal plants, and Malaysia, with database-specific syntax applied as detailed in Table 1. All retrieved records were imported into EndNote 21 (Clarivate Analytics) for reference management, and duplicate entries were removed. Titles and abstracts of the remaining records were independently screened by two authors based on predefined eligibility criteria. Full-text articles were subsequently assessed for inclusion, with any disagreements resolved through discussion and consensus.

### 2.2. Study Selection Criteria

Studies published between 1 January 2015 and 13 March 2025 were considered eligible. Inclusion criteria were as follows: (i) original research articles published in English; and (ii) studies investigating endophytic fungi isolated from medicinal plants collected in Malaysia. Exclusion criteria included non-original publications (e.g., reviews, book chapters, editorials, conference proceedings), studies conducted outside Malaysia, articles published in languages other than English, and studies involving non-plant hosts such as algae or seaweeds.

To enhance coverage beyond database indexing, backward citation tracking was performed by screening reference lists of included articles, and forward citation searches were conducted using Google Scholar to identify relevant studies published after the initial search. Any discrepancies identified during the selection process were resolved by consensus among the authors.

### 2.3. Data Extraction and Synthesis

Data from all the studies included were systematically extracted and organized using a standardized Microsoft Excel database. Extracted variables included publication year, host medicinal plant, plant tissue source, fungal taxa, identification methods, reported bioactivities, secondary metabolites, analytical techniques, study objectives, and key findings. Two authors independently reviewed and coded all eligible studies. The compiled dataset enabled structured qualitative synthesis and comparative analysis across studies, with particular attention to methodological trends, functional outcomes, and translational relevance. Discrepancies in data extraction or categorization were resolved through discussion.

## 3. Results

### 3.1. Identification of Studies via Databases and Registers

**Figure 1:**
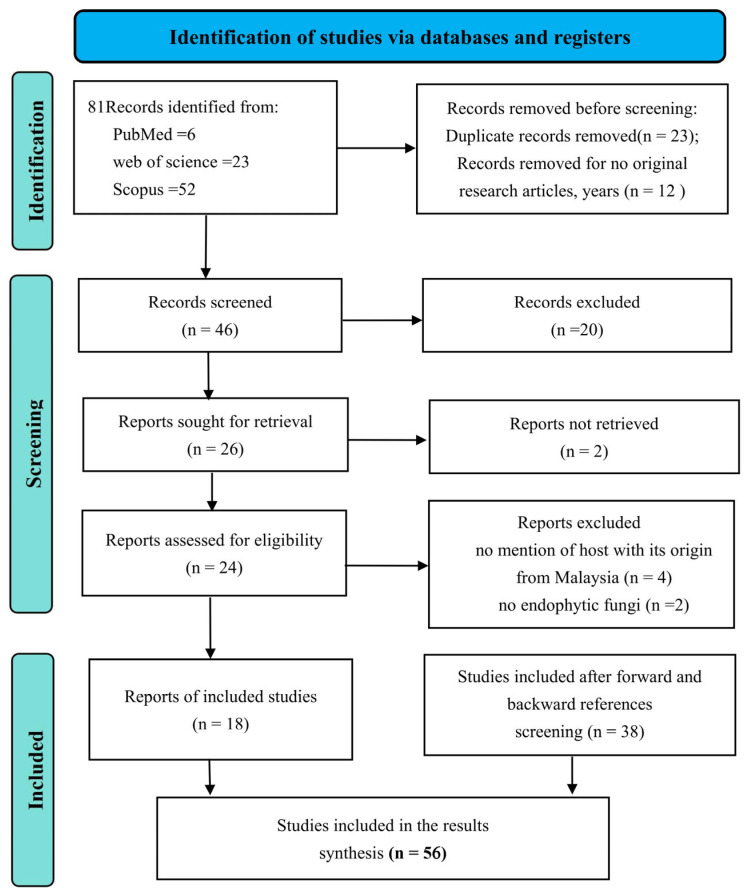
PRISMA flow diagram. Flow diagram illustrating the study identification, screening, eligibility assessment, and inclusion process used in this scoping review. The diagram was adapted from the PRISMA 2020 statement [10] and generated using the PRISMA 2020 flow diagram template distributed under the Creative Commons Attribution (CC BY 4.0) license.

The literature search yielded a total of 81 records from PubMed (*n* = 6), Web of Science (*n* = 23), and Scopus (*n* = 52). After removal of duplicates, 58 unique records were screened based on titles and abstracts, resulting in 26 articles assessed for full-text eligibility. Eighteen studies met the inclusion criteria following full-text evaluation. Additional forward and backward citation screening identified 38 further eligible studies. In total, 56 primary studies published between 2015 and March 2025 were included in the final synthesis (Figure 1). An overview of the included studies, including medicinal plant hosts, associated endophytic fungal taxa, investigated biological activities, and reported bioactive compounds, is summarized in Table 2.

### 3.2. Research Focus and Host-Endophyte Landscape

Across the 56 studies included in this scoping review, endophytic fungi were reported from 28 medicinal plant species traditionally used in Malaysian ethnomedicine. All documented hosts were terrestrial herbs, shrubs, or trees, with no aquatic or semi-aquatic plants represented. Sampling efforts primarily targeted aerial tissues, particularly leaves and stems, whereas roots, fruits, and reproductive organs were less frequently investigated.

The distribution of research attention across host plants was markedly uneven. *Ocimum sanctum* emerged as the most intensively studied host, appearing in 14 independent studies and accounting for approximately one quarter of the included literature [11,19,22,23,24,25,30,39,42,43,48,49,50,56]. A small group of additional medicinal plants, including *Swietenia macrophylla* (*n* = 5) [31,57,60,61,63], *Catharanthus roseus* [17,53,58], *Curcuma mangga* [12,21,27], and *Orthosiphon stamineus* (*n* = 4) [18,26,32,41], were investigated repeatedly, while the majority of host species (64.3%) were examined in only a single study, such as *Cymbopogon citratus* [13,65], *Centella asiatica* [44,66], *Melastoma malabathricum* [54], and *Pereskia bleo* [65]. This pattern indicates that host selection has been driven largely by research continuity and accessibility rather than by systematic or comparative bioprospecting strategies.

Geographically, sampling efforts were concentrated in Peninsular Malaysia, particularly in regions hosting major academic institutions such as Selangor and Penang. For example, *Swietenia macrophylla*, *Capsicum annuum*, *Oldenlandia diffusa*, and *Murraya koenigii* were primarily collected in Selangor [34,45,65], while *Ocimum sanctum* and *Orthosiphon stamineus* were extensively studied in Penang. Additional collections were reported from Kedah, Kelantan, Johor, Melaka, and Perak [14,15,55]. In contrast, East Malaysia was substantially underrepresented, with only a single study involving *Gynura procumbens* [40] and no reports from Sabah. This pronounced geographic bias suggests that large portions of Malaysia’s biodiversity-rich landscapes remain unexplored with respect to medicinal plant-associated endophytic fungi. These findings indicate that the current host–endophyte landscape reflects concentrated research effort rather than comprehensive coverage, highlighting substantial gaps in both host diversity and geographic representation.

### 3.3. Taxonomic Composition and Diversity Patterns of Endophytic Fungi

Taxonomic characterisation of endophytic fungi was explicitly addressed in 33.93% of the included studies (*n* = 19/56), collectively reporting 48 genera and 87 species (Supplementary Table S1). Across all studies, *Ascomycota* overwhelmingly dominated the recovered isolates, accounting for an estimated 90–95% of reported taxa, whereas *Basidiomycota* and *Mucoromycotina* were only sporadically detected. This skewed distribution is consistent with the use of culture-dependent isolation methods, which favour fast-growing ascomycetous fungi.

At higher taxonomic levels, isolates were primarily affiliated with the classes *Sordariomycetes* and *Dothideomycetes*, followed by *Eurotiomycetes*. Commonly reported orders included *Hypocreales*, *Glomerellales*, *Xylariales*, *Diaporthales*, and *Pleosporales*, encompassing families such as *Nectriaceae*, *Botryosphaeriaceae*, *Xylariaceae*, *Diaporthaceae*, and *Aspergillaceae*. In contrast, basidiomycetous endophytes, mainly from *Agaricomycetes*, were rarely encountered [52].

**Figure 2:**
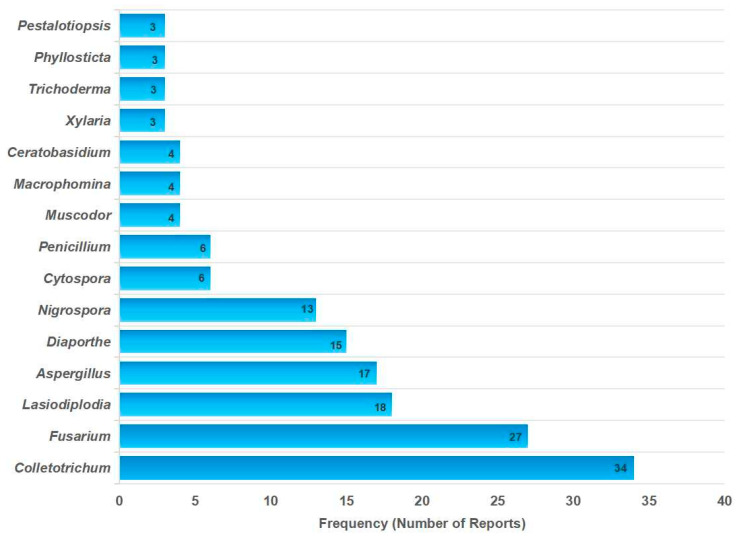
Distribution of endophytic fungal genera isolated from medicinal plants in Malaysia. Only genera reported more than twice in published studies between 1 January 2015 and March 2025 are shown.

At the genus level, a limited number of cosmopolitan taxa dominated the dataset. *Colletotrichum* represented approximately 16% of total isolates, followed by *Fusarium* (≈13%) and *Lasiodiplodia* (≈9%), with *Aspergillus*, *Diaporthe*, and *Nigrospora* also frequently reported (Figure 2). These genera were associated with multiple host plants and geographic locations, suggesting ecological generalism rather than host specificity.

Despite the apparent taxonomic breadth, species-level resolution remained limited. Most studies relied primarily on ITS sequencing, with relatively few employing multilocus approaches such as TEF1-α or β-tubulin markers [38]. Consequently, many isolates were reported only at the genus level or as ambiguous taxa (e.g., *Colletotrichum* sp., *Phomopsis* sp.), while sterile isolates were occasionally assigned only to higher taxonomic ranks [17,40,54]. These limitations underscore the constrained resolution of current taxonomic frameworks and highlight the need for integrated, high-resolution identification strategies to accurately characterise endophytic fungal diversity in Malaysian medicinal plants.

### 3.4. Bioactivities of Endophytic Fungi Associated with Malaysian Medicinal Plants

**Figure 3:**
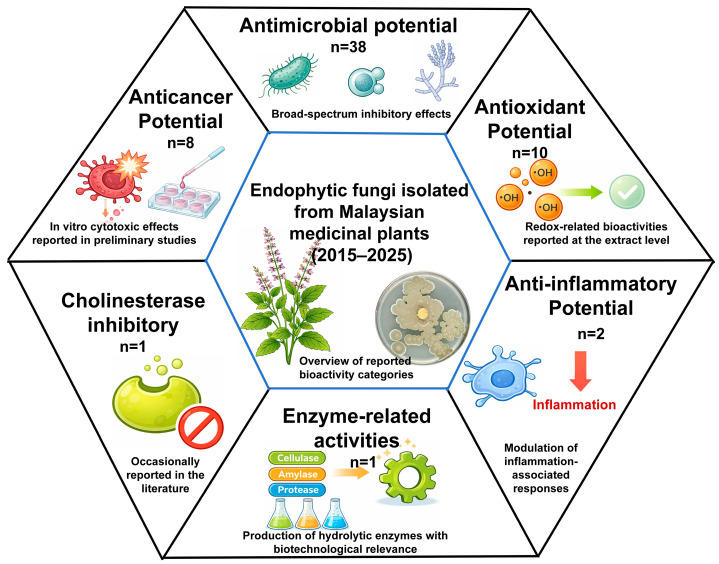
Distribution of reported bioactivity categories investigated in endophytic fungi isolated from Malaysian medicinal plants between January 2015 and March 2025. The hexagonal schematic summarizes six major bioactivity types, where *n* denotes the number of published studies reporting each bioactivity category.

Analysis of the included literature reveals that bioactivity evaluation constitutes the primary research focus in studies of endophytic fungi isolated from Malaysian medicinal plants, accounting for 87.93% of all included publications. These investigations were predominantly based on in vitro screening assays and focused mainly on antimicrobial, antioxidant, and cytotoxic (anticancer) activities, whereas other functional properties, such as anti-inflammatory, antidiabetic, and enzyme inhibitory activities, were comparatively underrepresented (Figure 3).

#### 3.4.1. Antimicrobial Potential of Endophytic Fungi

Antimicrobial activity emerged as the most extensively investigated bioactivity among endophytic fungi isolated from Malaysian medicinal plants, with 37 studies (69.81% of bioactivity-focused publications) evaluating antimicrobial potential. These studies targeted a broad range of microorganisms, including bacterial, yeast, and filamentous fungal pathogens. Bacterial targets predominated and commonly included *Bacillus cereus*, *Bacillus subtilis*, *Staphylococcus aureus*, *Salmonella typhimurium*, *Pseudomonas aeruginosa*, *Acinetobacter* spp., *Streptococcus faecalis*, *Klebsiella pneumoniae*, and *Shigella boydii*, whereas yeast and filamentous fungal targets were less frequently examined [14,57,59].

Across the reviewed literature, antimicrobial evaluation was largely based on conventional in vitro assays, including dual-culture assays, disc diffusion tests, agar plug methods, and minimum inhibitory or lethal concentration (MIC/MLC) determinations. In several studies, fermentation-based approaches were applied to enhance metabolite production prior to bioactivity assessment. For example, *Ceratobasidium ramicola* cultivated under submerged conditions yielded metabolites active against methicillin-resistant *Staphylococcus aureus* (MRSA) [16]. A limited subset of studies incorporated complementary assays, such as microbial kill curves or biofilm inhibition tests, providing additional functional insight beyond growth inhibition alone [14,31,42,50,56,57].

Large-scale screening studies further highlighted the high frequency of antimicrobial activity among Malaysian endophytic fungi. Bioactivity rates exceeding 90% were reported for isolates derived from *Curcuma mangga* [27], while earlier surveys demonstrated that more than 68% of screened isolates exhibited inhibitory activity against at least one test pathogen [67]. Enrichment strategies that incorporated host plant material into isolation media were also reported to enhance the recovery of bioactive endophytes, as exemplified by studies on *Orthosiphon stamineus*, in which up to 92% of isolates displayed antimicrobial activity [68].

Despite the predominance of extract-level screening, several studies reported notable antimicrobial potency against clinically relevant pathogens. For instance, *Phyllosticta fallopiae* L67 isolated from *Aloe vera* exhibited broad-spectrum antibacterial activity against diabetic wound pathogens, with MIC values ranging from 78.13 to 2500 μg/mL [14]. *Nigrospora sphaerica* demonstrated anti-biofilm activity against *Streptococcus mutans*, while *Ceratobasidium ramicola* showed measurable activity against MRSA [16]. In addition, *Buergenerula spartina* exhibited inhibitory and anti-biofilm effects against *S. aureus* and *B. cereus* [37].

Only a small number of studies progressed beyond crude extract screening to isolate and characterise individual bioactive compounds. Among these, *Penicillium purpurogenum* ED76 produced hydroxy-5-methoxy-hex-5-en-2,4-dione, which exhibited fungicidal activity against *Candida albicans* with a MIC of 3.1 μg/mL [60]. Polyketide and benzofuran derivatives isolated from *Phoma* sp. also demonstrated broad-spectrum antimicrobial effects [69]. In rare cases, preliminary mechanistic observations were reported, such as morphological damage to bacterial cell walls induced by extracts of *Lasiodiplodia pseudotheobromae* IBRL OS-64 [11]. These findings indicate that antimicrobial research on Malaysian medicinal plant-associated endophytic fungi has prioritised broad activity detection through extract-level screening, with comparatively limited advancement toward compound-resolved or mechanism-informed investigation.

#### 3.4.2. Antioxidant Potential of Endophytic Fungi

Antioxidant activity represented the second most frequently investigated bioactivity among endophytic fungi associated with Malaysian medicinal plants, reported in 10 studies (18.87% of bioactivity-focused publications). These studies encompassed a range of endophytic genera, including *Lasiodiplodia*, *Fusarium*, *Pseudopestalotiopsis*, *Diaporthe*, *Phomopsis*, and *Colletotrichum*, isolated from ethnomedicinal hosts such as *Ocimum sanctum*, *Dendrobium* spp., *Gynura procumbens*, and *Orthosiphon stamineus* [30,35,36,40,41,44,53,55,64,70].

Across the reviewed literature, antioxidant capacity was predominantly assessed using the 2,2-diphenyl-1-picrylhydrazyl (DPPH) radical scavenging assay, reflecting its widespread adoption as a rapid and cost-effective proxy for free radical neutralisation. In many studies, DPPH measurements were complemented by ferric reducing antioxidant power (FRAP) assays, total phenolic content (TPC), and total flavonoid content (TFC) analyses, providing a broader biochemical profile of extract-level antioxidant potential [30,36]. A smaller number of investigations combined antioxidant assays with antimicrobial testing, suggesting an interest in multifunctional bioactivity, although such correlations were generally exploratory.

Several endophytic fungi exhibited notable antioxidant performances. *Lasiodiplodia pseudotheobromae* IBRL OS-64, previously reported for antimicrobial activity, demonstrated strong DPPH radical scavenging capacity [30]. *Fusarium fujikuroi* isolated from *Dendrobium* spp. also showed promising antioxidant activity, highlighting the potential of orchid-associated endophytes as sources of antioxidative metabolites [35]. In one of the more systematic assessments, antioxidant activity across multiple endophytes was correlated with elevated phenolic and flavonoid contents, supporting the contribution of polyphenolic compounds to fungal-derived antioxidant effects. Antioxidant research on Malaysian medicinal plant-associated endophytic fungi remains largely confined to extract-level screening and chemical proxy assays. While these studies demonstrate recurrent antioxidant potential across diverse taxa, limited progression toward compound isolation or mechanism-informed evaluation constrains deeper insight into structure–activity relationships and translational relevance.

#### 3.4.3. Anticancer Potential of Endophytic Fungi

Anticancer activity was investigated in eight studies, accounting for 15.09% of the bioactivity-focused literature on endophytic fungi associated with Malaysian medicinal plants [13,33,34,47,58,59,61,65]. These studies primarily evaluated cytotoxic effects against a panel of human cancer cell lines, including A549 (lung carcinoma), HepG2 (hepatocellular carcinoma), HL-60 (promyelocytic leukaemia), HT-29 and HCT-116 (colorectal carcinoma), H103 (oral carcinoma), and Jurkat (T-cell leukaemia), reflecting an exploratory focus on broad antiproliferative potential [33,34,47].

Across the reviewed studies, cytotoxicity assessment relied predominantly on in vitro cell viability assays, most commonly the MTT assay. In several cases, fungal metabolite production was optimised using fermentation strategies such as one-factor-at-a-time (OFAT) optimisation or response surface methodology (RSM) [13,34]. A subset of investigations additionally evaluated enzymatic activities relevant to oncology, notably L-asparaginase production, recognising its established therapeutic role in the treatment of acute lymphoblastic leukaemia [13,35].

Notable anticancer-related findings were reported across multiple fungal genera. Early screening studies identified numerous endophytic isolates capable of producing L-asparaginase, with producers spanning *Colletotrichum*, *Fusarium*, *Phoma*, and *Penicillium*, underscoring the tractability of endophytes as microbial sources of clinically relevant enzymes [65]. Chemical investigations in later studies provided more direct links between endophytic fungi and anticancer-relevant metabolites. For example, an *Aspergillus* sp. isolate yielded rare diterpene pyrone compounds, including asperginols A and B, one of which exhibited moderate cytotoxic activity against HT-29 colorectal adenocarcinoma cells [47]. Subsequent work on the same strain expanded its chemical repertoire through the identification of additional alkaloid metabolites, including aspergillinines A–D, thereby broadening the accessible chemical space associated with this endophyte, even though these compounds did not display significant antiproliferative activity in standard assays [33]. Anticancer research on Malaysian medicinal plant-associated endophytic fungi remains limited in scope and largely confined to preliminary in vitro screening. While several studies demonstrate the capacity of endophytes to produce cytotoxic metabolites or therapeutically relevant enzymes, progression toward systematic structure–activity evaluation, mechanism-informed assays, and in vivo validation remains rare.

#### 3.4.4. Other Bioactivities of Endophytic Fungi

In addition to antimicrobial, antioxidant, and anticancer activities, a small subset of studies (five publications; 9.43% of bioactivity-focused research) reported other functional properties of endophytic fungi associated with Malaysian medicinal plants [15,17,29,32,38,45,46,53]. Although limited in number, these studies highlight the broader functional spectrum of endophytes beyond conventional drug-oriented bioactivity screening.

Reported activities included anti-inflammatory and enzyme inhibitory effects [29,32], which were primarily evaluated using extract-level assays. Anti-inflammatory potential was commonly assessed through nitric oxide inhibition assays, while enzyme-related activities included cholinesterase inhibition, suggesting possible neuroprotective relevance. However, these findings remain exploratory and were generally not supported by compound-level identification or mechanistic validation.

In addition to therapeutic applications, several studies documented industrially relevant enzymatic activities. Endophytic isolates from genera such as *Colletotrichum* and *Macrophomina* exhibited hydrolytic enzyme production, including cellulase, amylase, and protease activities, underscoring their potential utility in biotechnological processes [17].

A limited number of investigations also reported functional plasticity among endophytic fungi, with certain isolates displaying pathogenic behaviour under specific experimental conditions [38]. This duality highlights the dynamic ecological roles of endophytic fungi and underscores the need for cautious interpretation of bioactivity data.

### 3.5. Secondary Metabolites

More than 23% of publications reported the isolation and structural elucidation of secondary metabolites from endophytic fungi associated with Malaysian medicinal plants [11,13,14,18,32,33,34,37,40,41,47,58,59,64,66]. Across these studies, metabolite discovery predominantly followed conventional natural product workflows, involving single-strain cultivation under nutrient-rich conditions, organic solvent extraction, and chromatographic fractionation guided by bioactivity or chemical profiling [14,37]. Structural elucidation was primarily achieved using nuclear magnetic resonance (NMR) spectroscopy and mass spectrometry (MS), with gas chromatography–mass spectrometry (GC–MS) applied in selected cases, particularly for volatile metabolites [11,33].

**Figure 4:**
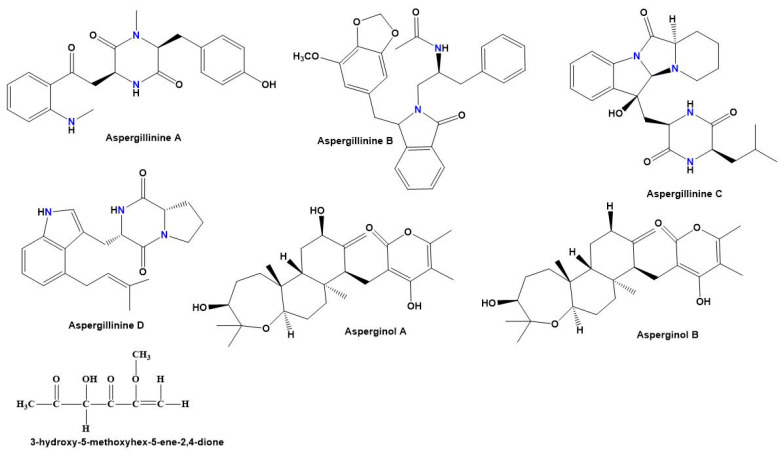
Selected examples of seven compounds reported as new natural products from endophytic fungi associated with Malaysian medicinal plants between 1 January 2015 and 13 March 2025. Nitrogen atoms are shown in blue.

These investigations reported 198 identified secondary metabolites spanning multiple structural classes, including alkaloids, terpenoids, polyketides, flavonoids, phenolic derivatives, and steroids (Supplementary Table S2). This apparent chemical breadth reflects the intrinsic biosynthetic capacity of medicinal plant-associated endophytic fungi. However, despite the number of reported compounds, only seven metabolites (3.5%) were described as new natural products, representing previously unreported molecular scaffolds (Figure 4) [18,33,47]. The low proportion of novel compounds indicates that much of the chemical output accessed to date corresponds to known or frequently rediscovered metabolites. This pattern likely reflects both pathway conservation among commonly isolated endophytic genera and methodological limitations associated with single-condition cultivation and activity-guided fractionation strategies. For clarity and consistency, the identified metabolites are grouped into major chemical categories commonly adopted in the source literature.

#### 3.5.1. Alkaloids

Alkaloid-type secondary metabolites were reported from a limited number of studies investigating endophytic fungi associated with Malaysian medicinal plants. The identified compounds span a broad structural range, from simple cyclic dipeptides to more complex indole- and ergoline-type alkaloids, reflecting the biosynthetic versatility of fungal endophytes.

Several reports highlighted the capacity of endophytic fungi to produce alkaloids typically associated with plant metabolism. For example, *Nigrospora sphaerica* isolated from *Catharanthus roseus* was reported to produce the bisindole alkaloid vinblastine, a well-established anticancer compound [18]. In addition, *Aspergillus* species exhibited pronounced alkaloid biosynthetic potential. In particular, *Aspergillus* sp. HAB10R12 isolated from *Garcinia scortechinii* yielded a series of diketopiperazine- and isoindolinone-type alkaloids, designated aspergillinines A–D, among which aspergillinine B represented a novel isoindolinone scaffold [33].

Other alkaloid classes reported included ergoline derivatives such as ergocornine, indole alkaloids such as psychotrine from *Buergenerula* sp. [37], and various cyclic dipeptides detected in unidentified sordariomycetous isolates [59]. Although many of these compounds correspond to known natural products, their repeated detection in endophytic fungi underscores the metabolic plasticity of these microorganisms and suggests either biosynthetic convergence or chemical mimicry with host plant pathways. Notably, alkaloid discovery remained sporadic and was rarely accompanied by systematic bioactivity evaluation or structure–activity analysis.

#### 3.5.2. Terpenoids

Terpenoid metabolites constituted another prominent class of secondary metabolites reported from Malaysian medicinal plant-associated endophytic fungi. Identified terpenoids encompassed mono-, sesqui-, di-, and triterpenoid derivatives, with chemical evidence derived primarily from GC–MS profiling and targeted compound isolation. Several studies reported the detection of volatile mono- and sesquiterpenes from endophytic fungi belonging to genera such as *Diaporthe*, *Colletotrichum*, and *Fusarium* isolated from *Gynura procumbens* [40]. These metabolites included compounds commonly associated with plant essential oils, such as terpinene, caryophyllene, eucalyptol, and globulol, suggesting biosynthetic convergence or close metabolic integration between endophytes and their host plants.

More detailed chemical investigations revealed structurally distinctive terpenoid-related metabolites. Notably, *Aspergillus* sp. HAB10R12 from *Garcinia scortechinii* produced a series of novel diterpene–polyketide hybrid compounds, termed aspergillipyrones A–D, and new sesquiterpenoid derivatives designated asperginols A–F [47]. Compounds such as aspergillipyrone A and asperginol A substantially expanded the known chemical diversity of fungal-derived terpenoids and highlighted the capacity of selected endophytic fungi to access uncommon terpenoid scaffolds. However, similar to alkaloid discovery, terpenoid investigations were largely confined to single-strain studies and lacked integration with genomic or biosynthetic analyses.

#### 3.5.3. Flavonoids and Phenolic Compounds

Flavonoid- and phenolic-related metabolites were reported in a limited number of chemically focused studies, primarily involving endophytic fungi isolated from polyphenol-rich medicinal plants. In these investigations, compound identification ranged from simple phenolic acids to structurally complex flavonoid scaffolds, indicating that selected endophytic fungi are capable of accessing plant-like phenolic chemical space.

One of the most comprehensive reports involved *Phyllosticta fallopiae* L67 isolated from *Aloe vera* [14], from which multiple subclasses of flavonoids and related phenolic compounds were identified, including flavone and isoflavone glycosides, prenylated flavonoids, stilbenoids, and chalcone derivatives. Among these, meliaedanoside A was characterised as a novel flavonoid glycoside, expanding the known structural diversity of flavonoid-associated metabolites reported from endophytic fungi.

In contrast, other studies reported the production of simpler phenolic acids and phenylpropanoid derivatives by endophytic species of *Diaporthe* and *Colletotrichum* isolated from *Orthosiphon stamineus* and *Gynura procumbens* [32,40]. These compounds, including gallic acid, caffeic acid, chlorogenic acid, cinnamic acid, and isoeugenol, are classically associated with plant secondary metabolism. Their detection in fungal cultures suggests potential biosynthetic convergence or close metabolic interaction between endophytes and their host plants. However, whether these metabolites arise from de novo fungal biosynthesis or from transformation of host-derived precursors remains unresolved.

Overall, evidence for flavonoid and phenolic metabolite production by endophytic fungi remains uneven and largely confined to extract- or single-isolate-level studies, with limited integration of biosynthetic, genomic, or mechanistic analyses.

#### 3.5.4. Polyketides and Non-Canonical Metabolites

Polyketide-derived metabolites represented one of the more chemically substantiated classes of secondary metabolites reported from Malaysian medicinal plant-associated endophytic fungi. Several studies achieved full structural elucidation of polyketide-type compounds, including both known frameworks and previously undescribed structures.

Notably, *Diaporthe* sp. ED2 isolated from *Orthosiphon stamineus* was reported to produce 3-hydroxy-5-methoxyhex-5-ene-2,4-dione, a novel polyketide-like metabolite featuring an uncommon linear scaffold [18]. In addition, known polyketides such as vermopyrone and anthraquinone derivatives were identified from endophytic species of *Aspergillus* and *Diaporthe*, illustrating the capacity of these genera to access diverse polyketide chemical space [41].

In addition to taxonomic canonical polyketide frameworks, several studies described the production of non-canonical or mixed-origin metabolites that blur the distinction between primary and secondary metabolism. For example, *Diaporthe fraxini* isolated from *O. stamineus* was reported to secrete metabolites such as melatonin, p-aminobenzoic acid, and orotic acid, reflecting metabolic versatility and potential cross-talk between primary metabolic pathways and secondary metabolite production [32]. In addition, *Fusarium proliferatum* isolated from *Cymbopogon citratus* was reported to produce L-asparaginase [13], a therapeutically relevant enzyme used in leukaemia treatment, extending the functional scope of endophytic fungi beyond small-molecule natural products.

Despite the apparent chemical and functional diversity, studies in this area remain predominantly descriptive. Most investigations focused on compound isolation and basic structural characterisation, with limited progression toward structure–activity relationship analysis, mechanistic validation, or systematic interrogation of biosynthetic pathways. Importantly, none of the reviewed studies established direct links between identified metabolites and their corresponding biosynthetic gene clusters or genomic architecture. This lack of integrative genomic context represents a critical constraint on understanding the genetic basis and regulatory logic of secondary metabolite biosynthesis in endophytic fungi.

## 4. From Descriptive Bioprospecting to an Actionable Genotype-to-Phenotype Discovery Workflow

This scoping review mapped the current research landscape of endophytic fungi associated with medicinal plants in Malaysia, with particular emphasis on reported biological activities, the extent of chemical characterization, and prevailing methodological trends. Rather than indicating a lack of biological potential, the accumulated evidence suggests that progress in the field has been constrained by a low-resolution, phenotype-first discovery paradigm. Although a broad range of bioactivities and metabolite classes has been reported, most studies remain focused on extract-level screening under routine cultivation conditions, with relatively limited advancement toward comprehensive chemical characterization, mechanistic understanding, and translational development.

A major constraint is the continued reliance on extract-level bioactivity screening under routine cultivation conditions, which preferentially accesses constitutively expressed metabolites while leaving many biosynthetic gene clusters (BGCs) unexpressed. Consequently, metabolite discovery remains biased toward known chemistry, and newly reported compounds are rarely investigated beyond preliminary structural characterization or bioactivity assessment. This limitation is compounded by the complete absence of whole-genome sequencing, BGC mining, and integrated multi-omics approaches in the studies reviewed, preventing robust links between genotype and chemical phenotypes from being established. As a result, strain selection remains largely empirical, biosynthetic potential remains underexplored, and reported bioactivities are seldom supported by mechanistic, pharmacological, or translational validation.

**Figure 5:**
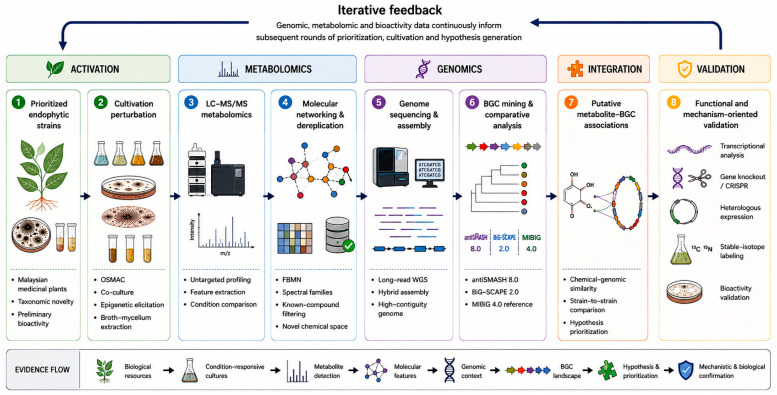
Proposed genotype-to-phenotype workflow for systematic natural-product discovery from endophytic fungi associated with Malaysian medicinal plants.

To address these limitations, we propose a practical genotype-to-phenotype workflow (Figure 5) for systematic natural-product discovery from endophytic fungi associated with Malaysian medicinal plants. The framework is designed as a modular and scalable pipeline that integrates cultivation perturbation, comparative LC–MS/MS metabolomics, genome sequencing, BGC analysis, metabolite–BGC linking, and mechanism-oriented validation. This design is consistent with recent fungal metabologenomics studies showing that paired genomic and metabolomic datasets can support the prioritisation of candidate metabolite–BGC relationships [71].

The workflow begins with strain prioritisation based on taxonomic distinctiveness, preliminary bioactivity, and experimental tractability. Prioritised strains are then subjected to cultivation-based perturbation strategies, including variation in fermentation conditions, co-culture, and chemical elicitation, to enhance the expression of otherwise silent or weakly expressed secondary metabolic pathways. Condition-responsive metabolite profiles are subsequently analysed using untargeted LC–MS/MS, feature-based molecular networking, and dereplication to identify responsive molecular families and prioritise potentially novel chemical space [72].

For high-priority strains, long-read or hybrid whole-genome sequencing can provide improved genomic continuity for BGC delineation. BGC prediction and comparative analysis can then be performed using updated platforms such as antiSMASH 8.0, BiG-SCAPE 2.0, and MIBiG 4.0, allowing predicted clusters to be compared with curated biosynthetic reference space [73]. Integration of metabolomic and genomic evidence can generate putative metabolite–BGC associations, which may then be evaluated through transcriptional analysis, targeted genetic perturbation, heterologous expression, stable-isotope labelling, and mechanism-oriented bioactivity assays. Stable-isotope approaches are particularly useful for strengthening links between natural products and biosynthetic pathways [74].

Importantly, this workflow should be viewed as iterative rather than linear. Evidence generated at each stage can feed back into strain re-prioritisation, cultivation redesign, and hypothesis refinement. By shifting discovery from descriptive extract screening toward evidence-linked chemical and biosynthetic interpretation, this framework provides a realistic route for more systematic exploration of Malaysia’s endophytic fungal diversity.

## 5. Conclusions

This scoping review demonstrates that medicinal plant-associated endophytic fungi in Malaysia constitute a taxonomically diverse and chemically promising reservoir of microbial biodiversity. The accumulated evidence highlights substantial potential to produce metabolites with antimicrobial, antioxidant, anticancer, and other biotechnologically relevant activities, reinforcing the importance of endophytic fungi as a largely underexplored source of natural products.

The review reveals a persistent gap between the documentation of biological activity and understanding of the underlying chemical, genetic, and functional mechanisms. Although numerous studies have reported bioactive extracts and metabolites, comparatively few have progressed toward comprehensive chemical characterization, biosynthetic interpretation, or mechanism-oriented validation. Consequently, much of the currently available evidence remains descriptive, limiting biological insight and translational value.

The principal challenge facing the field is therefore not the availability of promising fungal resources, but the analytical resolution at which they are investigated. Future progress will depend on strengthening the integration of chemical, biological, and molecular evidence to bridge the gap between phenotype and function. Advancing beyond descriptive bioprospecting toward a more mechanistic and predictive understanding of endophytic fungal systems will be essential for fully realizing the scientific, biotechnological, and pharmaceutical potential of Malaysia’s medicinal plant-associated mycobiota.

## Figures and Tables

**Table 1:** Search terms and database-specific search strategies used for literature retrieval.

Databases	Keywords Combination
PubMed	(“endophytic fungi” OR “endophyte” OR “endophytic mycobiota”) AND (“medicinal plant” OR “ethnomedicinal plant” OR “medicinal herb” OR “therapeutic plant”) AND (“Malaysia”)
Web of Science	(TS = (“endophytic fungi” OR “endophyte” OR “endophytic mycobiota”)) AND (TS = (“medicinal plant” OR “ethnomedicinal plant” OR “medicinal herb” OR “therapeutic plant”)) AND (CU = (“Malaysia”))
Scopus	(TITLE-ABS-KEY (“endophytic fungi” OR “endophyte” OR “endophytic mycobionts”) AND TITLE-ABS-KEY (“medicinal plant” OR “ethnomedicinal plant” OR “medicinal herb” OR “therapeutic plant”) AND AFFILCOUNTRY (“Malaysia”))

TS: topic search; ABS: abstract; KEY: keyword; AFFIL: Affiliation.

**Table 2:** Characteristics of the 56 studies included for data extraction and synthesis in this scoping review.

No.	Medicinal Plant	Endophytic Fungi	Reported Bioactivity	Key Findings	Bioactive Compounds	Ref.
1	*Ocimum sanctum*	*Lasiodiplodia pseudotheobromae* IBRL OS-64	Antibacterial	The ethyl acetate extract and its bioactive fraction exhibited broad-spectrum antibacterial activity against both Gram-positive and Gram-negative bacteria, including MRSA.	1,2-Benzenedicarboxylic acid, mono(2-ethylhexyl)ester	[11]
2	*Curcuma mangga* Valeton & Zijp	*Ceratobasidium ramicola* IBRLCM127	Antibacterial	*C. ramicola* IBRLCM exhibited prominent antibacterial activity against Gram-positive and Gram-negative bacteria.	Not reported	[12]
3	*Cymbopogon citratus*	*Fusarium proliferatum* (isolate CCH)	Enzyme production; Cytotoxic	The optimum condition for L-asparaginase production was determined to be glucose, L-asparagine, with around 5 days of incubation at 25 ± 2 °C, 120 rpm. This optimization increased enzyme yield from 16.75 ± 0.76 IU/mL to 22.42 ± 0.20 IU/mL. The crude enzyme exhibited notable cytotoxic activity against the leukemic Jurkat E6 cell line in a dose-dependent manner.	L-asparaginase	[13]
4	*Aloe vera*	*Phyllosticta fallopiae* L67	Antimicrobial; Antibiofilm	DCM extract showed significant antimicrobial activity. MIC and MLC ranged from 78.13 to 5000 mg/mL. The extract showed bactericidal effects, inhibited more than 82% of biofilm formation, and displayed dose-dependent toxicity in a zebrafish model. Bioassay-guided fractionation identified an active fraction (C6) with broad antimicrobial efficacy.	Kushenol I, kushenol M, kuwanon A, moracin C, ophiopogonanone B, 4′-methylpinosylvin and flavokawain A	[14]
5	*Garcinia atroviridis*	*Nigrospora sphaerica* *Lasiodiplodia theobromae* *Bjerkandera adusta* *Colletotrichum* sp. *Pestalotiopsis neglecta*	Antifungal; Biocontrol	A total of 111 endophytic fungi were isolated from *G. atroviridis* tissues, with 8 genera identified. Thirty-six isolates showed strong antagonistic activity, mainly through competition, mycoparasitism, and antibiosis, suggesting potential biocontrol mechanisms.	Not reported	[15]
6	*Curcuma mangga* Valeton & Zijp	*Ceratobasidium ramicola* IBRLCM127	Antibacterial (Anti-MRSA)	Submerged fermentation significantly enhanced the anti-MRSA activity of *C. ramicola* IBRLCM127. Under optimized culture conditions (host plant extract supplementation, dark incubation, 25 °C, two mycelial plugs, 120 rpm), the maximal anti-MRSA activity reached 42.50 ± 0.1 U/mL, representing an 11.72% increase compared with non-optimized conditions, while shortening the incubation period from 16 to 12 days.	Not reported	[16]
7	*Catharanthus roseus* (white and purple varieties)	*Colletotrichum* sp. *Macrophomina phaseolina* *Nigrospora sphaerica* *Fusarium solani*	Enzyme production	Five filamentous endophytic fungal strains isolated from leaves and roots exhibited extracellular hydrolytic enzyme activities. All strains produced cellulase; *Colletotrichum* sp. and *F. solani* produced amylase; only *F. solani* produced protease, supporting their role in host tissue colonisation and symbiotic interaction.	Not reported	[17]
8	*Orthosiphon stamineus* Benth	*Diaporthe* sp. ED2	Antifungal (anti-candidal)	A novel compound (HMD) was isolated with antifungal activity against *C. albicans*, showing a clear inhibition zone (14.7 ± 0.8 mm), low MIC (3.1 µg/mL), and fungicidal MFC (12.5 µg/mL), with activity comparable to voriconazole.	3-Hydroxy-5-methoxyhex-5-ene-2,4-dione	[18]
9	*Ocimum sanctum* Linn	*Lasiodiplodia pseudotheobromae* IBRL OS-64	Antibacterial (Anti-MRSA)	Optimized conditions significantly enhanced anti-MRSA activity from 21.22 to 50.00 U/mL, with a modest increase in fungal biomass. Notably, the increase in antibacterial activity was not strictly correlated with fungal growth.	Not reported	[19]
10	*Tamarindus indica* L.	31 species from 15 genera	Not evaluated	A total of 69 fungal isolates were identified, representing 31 species from 15 genera, with high diversity (Shannon–Wiener Index H’ = 3.083). *Colletotrichum* and *Diaporthe* were dominant, including commonly isolated endophytes and latent pathogens.	Not reported	[20]
11	*Curcuma mangga* Valeton & Zijp	*Ceratobasidium ramicola* IBRLCM127	Antifungal (anti-candidal)	The ethyl acetate extract demonstrated notable anti-*Candida* activity, with a 15.3 ± 0.6 mm inhibition zone and MIC/MYC values of 2.5 mg/mL. A concentration-dependent yeasticidal effect was observed in the time–kill assay. SEM and TEM analyses revealed severe cellular damage after 36 h of treatment at 2× MIC. In contrast, the methanolic extract showed no antifungal activity.	Not reported	[21]
12	*Ocimum sanctum* Linn	*Lasiodiplodia pseudotheobromae* IBRL OS-64	Antibacterial	The ethyl acetate extract showed strong antibacterial activity (zones: 14.2–26.0 mm), with MICs of 62.5–500 µg/mL and MBCs of 125–2000 µg/mL (bactericidal). Gram-positive bacteria were more sensitive. SEM revealed membrane collapse, crumpling, and cavities in treated cells.	Not reported	[22]
13	*Ocimum sanctum* Linn	*Colletotrichum* sp. IBRL OS-27, *Colletotrichum* sp. IBRL OS-39, *Lasiodiplodia* sp. IBRL OS-64 *Aspergillus* sp. IBRL OS-65, *Aspergillus* sp. IBRL OS-82, *Muscodor* sp. IBRL OS-94, and OS-98	Antifungal	*Muscodor* OS-94/98 showed volatile antifungal activity (≤53.6%); *Colletotrichum* OS-39 up to 62.5%. *Dichloromethane* extracts were fungicidal (MFC/MIC ≤ 4) against *C. albicans*/*C. utilis*, fungistatic on others. SEM revealed membrane damage and cell shrinkage.	Not reported	[23]
14	*Ocimum sanctum* Linn	*Lasiodiplodia pseudotheobromae* IBRL OS-64	Antibacterial (including anti-MRSA)	Ethyl acetate extract showed strong antibacterial activity (zones: 20.0–31.3 mm Gram-positive; 10.3–20.1 mm Gram-negative), with lower MIC/MBC than methanol extract. Gram-positive bacteria were more susceptible. SEM showed cell wall rupture and cytoplasmic leakage in MRSA and *P. aeruginosa*. Both extracts were bactericidal (MBC/MIC ≤ 4).	Not reported	[24]
15	*Ocimum sanctum* Linn	*Lasiodiplodia pseudotheobromae* IBRL OS-64 *Muscodor* sp. IBRL OS-94	Anti-yeast (anti-*Candida*)	Both extracts, especially ethyl acetate, showed strong anti-yeast activity against *C. albicans* (most sensitive); *C. neoformans* was resistant. MIC/MYC of Muscodor sp. extract: 250–500/1000–4000 µg/mL (yeasticidal). SEM/TEM showed shrinkage and cytoplasmic leakage. Time–kill assay confirmed dose-dependent yeast-static to yeasticidal effects.	Not reported	[25]
16	*Orthosiphon stamineus* Benth	*Penicillium minioluteum* ED24	Antibacterial (anti-MRSA)	Disc diffusion showed 18.7 ± 0.5 mm inhibition (comparable to chloramphenicol). MIC/MLC: 31.25/125 µg/mL (bactericidal, MLC/MIC ≤ 4). Time–kill: 99.9% bacterial reduction. Brine shrimp assay: LC_50_ = 1.48 mg/mL (acute), 1.13 mg/mL (chronic), indicating non-toxicity (LC_50_ > 1.0 mg/mL).	Not reported	[26]
17	*Curcuma mangga* Valeton & Zijp	*Ceratobasidium ramicola* IBRLCM127	Antibacterial (anti-MRSA)	MIC/MBC = 500 µg/mL (bactericidal). At 2× MIC, MRSA was completely killed within 44 h (time- and dose-dependent). SEM showed progressive damage: cavities (12 h), clumping (24 h), and full lysis (36–48 h). First report of *C. ramicola* with anti-MRSA activity.	Not reported	[27]
18	*Cassia siamea* Lamk	*Aspergillus flavus* IBRL-C8	Antibacterial (anti-MRSA)	Ethyl acetate extract showed strong anti-*Candida* activity (28.3 ± 2.4 mm); methanol inactive. MIC/MYC = 1.00/4.00 mg/mL (fungicidal). Yeastostatic at ½ MIC, yeasticidal at MIC/2MIC. SEM/TEM revealed invaginations, cavitation, membrane disruption, and cytoplasmic disorganization in *C. albicans* after 36 h at MIC.	Not reported	[28]
19	*Cinnamomum porrectum*	5 fungal isolates (*Cytospora rhizophorae*)	Anti-inflammatory (neuroinflammation)	At 0.1 mg/mL, all five extracts significantly suppressed NO production (57–74%) without cytotoxicity; 1.0 mg/mL reduced cell viability. IL-6 and TNF-α were markedly reduced (up to 87–89%) in LPS-stimulated cells. No significant effects on IL-10, IL-12p70, IFN-γ, or MCP-1. CD40 expression was unaffected in stimulated microglia.	Not reported	[29]
20	*Ocimum sanctum* L.	*Lasiodiplodia pseudotheobromae* IBRL OS-64	Antioxidant; antibacterial; cytotoxicity	Fraction F5 showed stronger antioxidant activity (EC_50_ = 208.1 µg/mL) than crude (441.6 µg/mL), though both were weaker than quercetin. Phenolic content was low. MICs: 62.5–250 µg/mL (Gram-positive), 250–500 µg/mL (Gram-negative); MBCs: 125–2000 µg/mL. Fraction was non-toxic; crude showed mild chronic toxicity.	Not reported	[30]
21	*Swietenia macrophylla* (Mahogany)	*Nigrospora sphaerica* CL-OP30	Antibacterial; antibiofilm	*N. sphaerica* CL-OP30 extract inhibited *S. mutans* biofilm formation by 88.81% at 10 mg/mL. Microscopy showed matrix disintegration and cell deformation. The extract interfered with adherence via non-bactericidal action, likely by targeting exopolysaccharide synthesis.	Not reported	[31]
22	*Orthosiphon stamineus* (Misai Kucing)	*Diaporthe fraxini*	Cholinesterase inhibitory activity	Rosmarinic acid-supplemented media altered metabolite profiles. The extract (E-RA) showed enhanced AChE and BuChE inhibition. Key metabolites included kynurenic acid, caffeic acid, and gallic acid. PCA and clustering clearly separated treatment groups.	Kynurenic acid, caffeic acid, gallic acid, chlorogenic acid, cinnamic acid, 3-hydroxyanthranilic acid, melatonin	[32]
23	*Garcinia scortechinii*	*Aspergillus* sp. HAB10R12	No significant cytotoxicity	Four new alkaloids (aspergillinine A–D) and four known diterpene pyrones were isolated. Aspergillinine A featured N-methyl kynurenine; B had a novel isoindolinone core. All compounds showed no significant cytotoxicity (IC_50_ > 30 μM).	Not reported	[33]
24	*Oldenlandia diffusa*	*Colletotrichum gloeosporioides* (strain OD3)	Enzyme production; anticancer activity	Optimized conditions yielded purified L-asparaginase (255.02 IU/mg, 6.12-fold, 34.63% recovery). Enzymes are likely to be tetrameric. Greater cytotoxicity on Jurkat cells (IC_50_: 46.36 mg/mL) than H103 (125.56 mg/mL).	L-asparaginase	[34]
25	*Dendrobium* spp.	*Fusarium fujikuroi* *Fusarium proliferatum* *Fusarium oxysporum* *Fusarium verticillioides* *Trichoderma asperellum* *Daldinia eschscholtzii* *Nigrospora pyriformis*	Antioxidant activity; L-asparaginase production	A total of 89.7% of isolates produced L-asparaginase. *F. fujikuroi* (D1) had highest antioxidant activity; *D. eschscholtzii* (D14) showed highest enzyme activity. Some strains had dual antioxidant and enzymatic properties.	Not reported	[35]
26	*Pereskia bleo* *Murraya koenigii Oldenlandia diffusa* *Cymbopogon citratus*	*Pseudopestalotiopsis theae* *Fusarium solani* *Xylaria venustula* *F. proliferatum* *Colletotrichum boninense* *C. gloeosporioides* *C. siamense*	Antioxidant activity; antimicrobial activity; anti-candidal activity	Crude extracts from cultures exposed to green and red light exhibited stronger antibacterial activity (ZOI up to 38.30 ± 2.90 mm; MIC as low as 0.0196 mg/mL) and improved anti-Candida albicans activity in selected isolates, compared with white light and dark controls.	Not reported	[36]
27	*Cymbidium* spp.	*Buergenerula spartinae*	Antibacterial activity	Fractions 2 and 4 exhibited potent antibacterial activity against Staphylococcus aureus and Bacillus cereus, with MIC values as low as 0.078 mg/mL and 0.313 mg/mL, respectively. LC-MS profiling indicated that antibacterial activity was associated with specific fractions rather than crude extracts,	Kaempferol 3-p-coumarat; 6-methoxy naphthaleneacetic acid; kanzonol N; 3-butylidene-7-hydroxyphthalide; benoxinate; pyropheophorbide A; (−)-ormosanine; levofuraltadone; 3-α(S)-strictosidine; 5′-hydroxystreptomycin; hinokitiol glucoside; N-undecylbenzenesulfonic acid	[37]
28	*Calamus castaneus*	*Colletotrichum boninense* *C. fructicola* *C. cliviae* *Diaporthe hongkongensis* *D. arengae* *Neopestalotiopsis saprophytica* *N. formicarum* *Fusarium solani* *F. oxysporum*	Pathogenicity/virulence assessment	Ten endophytic fungi isolated from rattan spines exhibited varying degrees of virulence against leaves of *C. castaneus*, bertam (*Eugeissona* sp.), oil palm (*Elaeis guineensis*), mango (*Mangifera indica*), and fruits of chilli, tomato, and banana. Most isolates caused moderate to very high disease severity on wounded tissues, while reduced virulence was observed on unwounded tissues.	Not reported	[38]
29	*Ocimum sanctum*	*Lasiodiplodia pseudotheobromae* IBRL OS-64	Antibacterial activity	Ethyl acetate extract was more active than methanol, most effective against *Exiguobacterium profundum* (MIC/MBC: 125 µg/mL). SEM showed cell wall damage. Low toxicity (LC_50_ > 1000 µg/mL).	Not reported	[39]
30	*Gynura procumbens*	*Diaporthe hongkongensis Phomopsis* sp. *Colletotrichum truncatum Mycoleptodiscus indicus* *Diaporthe longicolla, Macrophomina phaseolina Beltraniella portoricensis* *C. asianum* *C. brevisporum, Fusarium incarnatum* *C. gloeosporioides* *Pestalotiopsis* sp.	Antibacterial activity; antioxidant activity	Extracts inhibited *S. aureus*, *P. aeruginosa*, MRSA, *E. coli*, *S. typhi* with MIC and MBC = 5000 µg/mL. Ethyl acetate extract of *M. phaseolina* SN6 showed DPPH inhibition of 86.6%, IC_50_ = 104.25 ± 18.51 µg/mL, and FRAP = 239.9 mg Fe(II)/g; methanolic extract of *M. indicus* SN4 showed highest scavenging (50.0%) and FRAP = 44.7 mg Fe(II)/g. TPC/TFC: highest ethyl acetate TPC from *C. gloeosporioides* SN11 (87.0 mg GAE/g); highest ethyl acetate TFC from *M. phaseolina* SN6 (122.8 mg QCE/g); highest methanolic TPC/TFC from M. indicus SN4 (35.0 mg GAE/g; 60.4 mg QCE/g).	Isoelemicin, eucalyptol, terpinen-4-ol, oleic acid, β-pinene	[40]
31	*Orthosiphon stamineus* (Java tea)	*Diaporthe fraxini* ED2	Antioxidant activity	DFS (RA-supplemented) enhanced TPC, TFC, and antioxidant activity (DPPH IC_50_ = 7.11 µg/mL). Metabolomics revealed 15 key bioactive compounds with antioxidant, antimicrobial, and neuroactive properties.	Hexamethylquercetagetin; thioquinolactobactin; vermopyrone; aculeatin A; toxicol B; 3-acetyl-4-hydroxy-6-methyl-2H-pyran-2-one; 5-acetyl-2-hydroxybenzaldehyde; anticapsin	[41]
32	*Ocimum sanctum*	*Lasiodiplodia pseudotheobromae* IBRL OS-64	Antibacterial activity (anti-MRSA)	Incorporation of *Ocimum sanctum* extract significantly enhanced the anti-MRSA activity of the endophytic fungus, as demonstrated by increased inhibition zone, lower MIC/MBC values, and stronger bactericidal effects.	Not reported	[42]
33	*Ocimum sanctum*	*Muscodor* sp. IBRL OS-94	antimicrobial activity	Extracellular extract exhibited stronger antimicrobial activity than intracellular extract. Effective against Gram-positive and selected Gram-negative bacteria, as well as yeasts and some fungi. SEM showed severe damage to microbial cell walls and membranes. Extract demonstrated both bactericidal and fungistatic effects depending on the organism.	Not reported	[43]
34	*Centella asiatica*	*Aspergillus austroafricanus* MB1 *Aspergillus oryzae* MM13	Antioxidant activity	All isolates showed antioxidant activity; *A. oryzae* MM13 (IC_50_ = 10.29 ppm) and *A. austroafricanus* MB1 (IC_50_ = 12.08 ppm) were most potent; GC-MS identified flavonoids, fatty acids, and carboxylic acids; Malaysian isolates yielded more antioxidant spots on TLC.	2,3-Dihydro-3,5-dihydroxy-6-methyl-4H-pyran-4-one	[44]
35	*Capsicum annuum* L.	*Trichoderma reesei* *Hypoxylon* sp. *Aspergillus awamori* *Aulographum hederae* *Bipolaris sorokiniana* *Patellaria atrata* *Aspergillus novofumigatus*	Antifungal/biocontrol activity	A total of 14 fungal morphotypes were identified, 11 confirmed by sequencing (*Ascomycota*); *Hypoxylon* sp. (F2) and *T. reesei* (F4) showed strong antagonistic activity (87.21% and 76.26% inhibition, respectively); Potential candidates for biocontrol application.	Not reported	[45]
36	*Ficus carica*	11 unidentified endophytic fungal isolates	Biocontrol activity	Isolates S2-1 and R3-4 exhibited the strongest antagonistic activity against *Ganoderma boninense*, *Magnaporthe oryzae*, and *Fusarium verticillioides*, with percentage inhibition of radial growth (PIRG) values exceeding 20–30%, mediated via competition and antibiosis mechanisms. In addition, several isolates displayed phosphate-solubilizing ability on Pikovskaya’s medium, with isolate S2-4 showing the highest phosphate solubilization index (PSI = 3.02 ± 0.05),	Not reported	[46]
37	*Garcinia scortechinii*	*Aspergillus* sp. HAB10R12	Cytotoxic activity	Two new compounds (asperginols A and B) identified; Four known analogues (asperginols C–F) structurally revised; Compounds showed unusual 7/6/6 tricyclic diterpene core with trans–syn–trans configuration; VCD and DFT clarified side-chain configurations; Compound 6 exhibited moderate cytotoxicity against HT-29 cells (IC_50_ = 7.6 μM).	Asperginol F	[47]
38	*Ocimum sanctum* Linn.	*Lasiodiplodia pseudotheobromae* IBRL OS-64	Antibacterial activity	Moderate inhibition zone (12.0 ± 0.4 mm); MIC was 250 µg/mL while MBC was 500 µg/mL; SEM revealed pits and lysis of cells.	Not reported	[48]
39	*Ocimum sanctum* Linn.	*Lasiodiplodia pseudotheobromae* IBRL OS-64	Anti-candidal activity	The MIC was 1 mg/mL while MYC was 2 mg/mL; Dose-dependent time–kill; SEM/TEM showed structural damage to *C. albicans*.	Not reported	[49]
40	*Ocimum sanctum* Linn.	*Lasiodiplodia pseudotheobromae* IBRL OS-64	Antibacterial and antibiofilm activities	The ethyl acetate extract showed the highest inhibition zone (20.3 mm), with MIC and MBC values of 125 and 250 µg/mL, respectively. Biofilm inhibition was 69.12% for initial and 58.70% for pre-formed biofilms, indicating strong antibacterial and antibiofilm activities against *Y. enterocolitica*.	Not reported	[50]
41	*Ocimum sanctum* Linn.	*Lasiodiplodia pseudotheobromae* IBRL OS-64	antibacterial activity	The fungal crude extracts demonstrated favourable antibacterial activity toward both test bacteria and produced an inhibition zone ranging from 16.0 to 21.2 mm. The MIC and MBC values of the fungal crude extract toward *S. mutans* and *S. agalactiae* were determined and the results showed that the MIC and MBC values were in the range of 125–500 μg/mL and 125–1000 μg/mL, respectively. The time–kill study suggested that the ethyl acetate crude extract possessed bactericidal effect in a concentration- and time-dependent manner.	Not reported	[51]
42	*Pandanus* sp., *Alpinia* sp.	*Colletotrichum* sp. (P1), *Lentinus* sp. (A1), *Zygomycota* sp. (P2)	Not evaluated	Three endophytic fungi were successfully isolated. *Pandanus* sp. exhibited a higher colonization frequency (16.7%) compared to *Alpinia* sp. (8.3%), suggesting greater fungal diversity or endophytic association in *Pandanus* sp.	Not reported	[52]
43	*Catharanthus roseus*	*Nigrospora sphaerica Macrophomina phaseolina Fusarium solani Colletotrichum gloeosporioides*	Antioxidant activity	*Nigrospora sphaerica* (CSE) demonstrated the highest TPC, TFC, and FRAP values, although its DPPH and FCA scavenging activities remained low, indicating limited free radical neutralization capacity.	Not reported	[53]
44	*Melastoma malabathricum* L.	*Ascomycota* & *Zygomycota*	Antifungal activity	Isolate SSM1 exhibited the highest antifungal inhibition (57.89%). Leaves showed the greatest colonization frequency (38.9%), highlighting them as a key source of diverse and potentially bioactive endophytes.	Not reported	[54]
45	*Rhizophora mucronata*	*Pestalotiopsis*, *Alternaria*, *Cladosporium*, *Fusarium lateritium*, *Nigrospora oryzae*, *Phoma* sp., *Xylaria* sp.	Antagonistic activity; antibacterial activity; antioxidant activity	*Xylaria* sp. demonstrated over 90% antioxidant activity. Several fungal isolates exhibited antagonistic activity against *F. solani*, suggesting their potential as biocontrol agents in mangrove and agricultural ecosystems.	Not reported	[55]
46	*Ocimum sanctum* Linn.	*Lasiodiplodia pseudotheobromae* IBRL OS-64	Antibacterial activity (anti-MRSA)	The extract showed a bactericidal effect with MBC/MIC ratio of 2. The most effective killing was observed at 2× MIC within 36 h. SEM analysis confirmed membrane disruption in MRSA cells.	Not reported	[56]
47	*Swietenia macrophylla*	*Aspergillus* sp. IBRL MP15 CCL	Antimicrobial activity (antibacterial and anti-yeast)	The extract demonstrated broad-spectrum antimicrobial activity with MIC values ranging from 250 to 4000 µg/mL. Strongest effects were observed against *S. faecalis* and *C. utilis*, indicating its potential as a bioactive agent.	Not reported	[57]
48	*Catharanthus roseus*	*Nigrospora sphaerica*	Anticancer/cytotoxic activity	The endophyte produced higher levels of vinblastine than the host plant. It exhibited stronger cytotoxicity and completed the production cycle within one month, compared to one year in *C. roseus*, suggesting significant biotechnological potential.	Vinblastine	[58]
49	*Strobilanthes crispus*	*Sordariomycetes* sp. (PDA)BL3 and (PDA)BL5	Antimicrobial activity; anticancer activity	(PDA)BL3 showed antimicrobial activity; (PDA)BL5 was cytotoxic to five cancer cell lines; GC-MS detected Pyrrolo[1,2-a] pyrazine-1,4-dione.	Pyrrolo[1,2-a]pyrazine-1,4-dione, hexahydro-3-(2-methylpropyl)	[59]
50	*Swietenia macrophylla*	*Penicillium purpurogenum* ED76	Antimicrobial activity (against bacteria and yeasts)	Broad-spectrum antimicrobial activity (Gram-positive/negative, yeasts); MIC range: 125–1000 µg/mL; MLC range: 1000–4000 µg/mL; SEM revealed *S. aureus* cell wall invagination and lysis; Major compound: stigmasterol (45.30%)	Stigmasterol	[60]
51	*Swietenia macrophylla*	*Nigrospora sphaerica* CL-OP30	Antibacterial activity	2× MIC extract killed 99.9% of cells; SEM/TEM revealed cell membrane disruption.	Not reported	[61]
52	*Psilotum nudum*	*Aspergillus niger, A. terreus* *Bipolaris* sp., *Coccidioides immitis*, *Paracoccidioides brasiliensis*, *Verticillium* sp. *A. flavus*, *Scedosporium apiospermum*	Not evaluated	Rhizoid had highest colonization; *Aspergillus* spp. dominant; hyphal coils visible.	Not reported	[62]
53	*Swietenia macrophylla* King	*Nigrospora sphaerica* CL-CP30	Antibacterial activity (foodborne pathogens)	Strong antibacterial activity against Gram-positive bacteria (*B. cereus*, *B. subtilis*, *B. spizizenii*, and *S. aureus*); No inhibition against Gram-negative bacteria or yeast; MIC for *S. aureus* = 93.75 µg/mL; MBC = 750 µg/mL; SEM revealed cell wall shrinkage, rupture, and surface roughening.	Not reported	[63]
54	*Cymbidium* sp.	*Fusarium fujikuroi*, *F. incarnatum*, *F. proliferatum*, *F. oxysporum* *Lasiodiplodia theobromae*, *Nigrospora oryzae*, *Buergenerula spartinae*	Antioxidant activity; L-asparaginase production	All 30 isolates showed antioxidant activity (45.28–76.4% RSA). *L. theobromae* had the strongest antioxidant effect (IC_50_ = 5.75 mg/mL; AEAC = 12.17 mg/g). 16 isolates (53.33%) were positive for L-asparaginase activity. *B. spartinae* showed the highest L-asparaginase activity (1.736 unit/mL).	Not reported	[64]
55	*Pereskia bleo* *Murraya koenigii Oldenlandia diffusa* *Cymbopogon citratus*	*Colletotrichum*, *Fusarium*, *Penicillium*, *Phoma*, and unidentified *Ascomycota* and *Dothideomycetes*	Enzyme activity	355 endophytes were isolated; 203 from *Pereskia bleo*; 25 morphotypes were L-asparaginase producers; highest activity from *Oldenlandia diffusa* isolate (ODL4 = 0.025 μM mL^−1^ min^−1^); Endophytes belonged to *Colletotrichum*, *Fusarium*, *Penicillium*, *Phoma*, *Ascomycota*, and *Dothideomycetes*.	Not reported	[65]
56	*Centella asiatica*	23 fungi endophytic	Not reported	A total of 145 endophytic fungal isolates were obtained from stolons, leaves, roots, and petioles. These were grouped into 23 morphotaxa (Malaysia). Phylogenetic analysis identified various species, predominantly from the genera *Fusarium*, *Aspergillus*, *Colletotrichum*, and *Phoma*. *Fusarium* (Nectriaceae) was the most dominant genus. Most isolates belonged to *Ascomycota* and *Basidiomycota*.	Not reported	[66]

## Data Availability

Data availability is not applicable to this article as no new data were created or analysed in this study. All data, in the form of previously published studies, that support the findings of this review are comprehensively cited in the reference list.

## Supplementary Materials

The following supporting information can be downloaded at: https://www.mdpi.com/article/10.3390/jof12070494/s1, Table S1: Endophytic fungi isolated from Malaysian medicinal plants reported between 1 January 2015 and 13 March 2025; Table S2: Natural Products Derived from Malaysian Medicinal Plants from 1 January 2015 to 13 March 2025.

## Abbreviations

The following abbreviations are used in this manuscript:

AChE	Acetylcholinesterase
AEAC	Ascorbic Acid Equivalent Antioxidant Capacity
BuChE	Butyrylcholinesterase
CD40	Cluster of Differentiation 40
CSE	Crude Secondary Extract
DCM	Dichloromethane
DFT	Density Functional Theory
DFS	Rosmarinic Acid-Supplemented Dark Fermentation System
DPPH	2,2-Diphenyl-1-picrylhydrazyl
EC_50_	Half Maximal Effective Concentration
FCA	Ferric Chelating Activity
FRAP	Ferric Reducing Antioxidant Power
GC-MS	Gas Chromatography–Mass Spectrometry
HT-29	Human Colorectal Adenocarcinoma Cell Line
IC_50_	Half Maximal Inhibitory Concentration
IFN-γ	Interferon-gamma
IL-6	Interleukin-6
IL-10	Interleukin-10
IL-12p70	Interleukin-12p70
L-NAME	Nω-Nitro-L-arginine Methyl Ester
LC-MS	Liquid Chromatography–Mass Spectrometry
MBC	Minimum Bactericidal Concentration
MCP-1	Monocyte Chemoattractant Protein-1
MFC	Minimum Fungicidal Concentration
MIC	Minimum Inhibitory Concentration
MLC	Minimum Lethal Concentration
MRSA	Methicillin-Resistant Staphylococcus aureus
MYC	Minimum Yeasticidal Concentration
NO	Nitric Oxide
PCA	Principal Component Analysis
PDA	Potato Dextrose Agar
PSI	Phosphate Solubilization Index
RSA	Radical Scavenging Activity
SEM	Scanning Electron Microscopy
TEM	Transmission Electron Microscopy
TFC	Total Flavonoid Content
TLC	Thin Layer Chromatography
TNF-α	Tumor Necrosis Factor-alpha
TPC	Total Phenolic Content
VCD	Vibrational Circular Dichroism
ZOI	Zone of Inhibition
