# Supplementary material for: Untapped Mycobiota: A Scoping Review of Endophytic Fungi in Medicinal Plants from Malaysia
Source: J Fungi (Basel). 2026 Jul 5;12(7):494. doi: 10.3390/jof12070494 (PMC13412894; doi:10.3390/jof12070494)
Supplement: Supplementary file 1 [file jof-12-00494-s001.zip › jof-4382041-supplementary.pdf]

Supplementary Table S1. Endophytic fungi isolated from Malaysian medicinal plants reported between 1 January 2015 and 13 March 2025

No.	Fungi	Medical plant	Parts	Identification method	Reference
1	*Lasiodiplodia pseudotheobromae IBRL OS-64*	*Ocimum sanctum*	leaf	morphological characteristics and ITS	[1]
2	*Ceratobasidium ramicola IBRLCM127*	*Curcuma mangga Valeton & Zijp*	rhizome	morphological characteristics and ITS	[2]
3	*Fusarium proliferatum*（*isolate CCH*）	*Cymbopogon citratus*	leaf	morphological characteristics and ITS	[3]
4	*Phyllosticta fallopiae L67*	*Aloe vera*	stem	morphological characteristics and ITS	[4]
5	*Colletotrichum gloeosporioides*	*Garcinia atroviridis*	stem	morphological characteristics and ITS	[5]
6	*Nigrospora sphaerica*	*Garcinia atroviridis*	stem	morphological characteristics and ITS	
7	*Lasiodiplodia theobromae*	*Garcinia atroviridis*	stem	morphological characteristics and ITS	
8	*Bjerkandera adusta*	*Garcinia atroviridis*	stem	morphological characteristics and ITS	
9	*Colletotrichum sp.*	*Garcinia atroviridis*	stem	morphological characteristics and ITS	
10	*Diaporthe sp.*	*Garcinia atroviridis*	stem	morphological characteristics and ITS	
11	*Pestalotiopsis neglecta*	*Garcinia atroviridis*	stem	morphological characteristics and ITS	
12	*Ceratobasidium ramicola IBRLCM127*	*Curcuma mangga Valeton & Zijp*	rhizome	morphological characteristics and ITS	[6]
13	*Colletotrichum sp.*	*Catharanthus roseus*	leaf and root	morphological characteristics and ITS	[7]
14	*Macrophomina phaseolina*	*Catharanthus roseus*	leaf and root	morphological characteristics and ITS	
15	*Nigrospora sphaerica*	*Catharanthus roseus*	leaf and root	morphological characteristics and ITS	
16	*Fusarium solani*	*Catharanthus roseus*	leaf and root	morphological characteristics and ITS	
17	*Diaporthe sp. ED2*	*Orthosiphon stamineus Benth*	leaf	morphological characteristics and ITS	[8]
18	*Lasiodiplodia pseudotheobromae IBRL OS-64*	*Ocimum sanctum Linn*	leaf	morphological characteristics and ITS	[9]

19	*Colletotrichum gloeosporioides*	*Tamarindus indica L*	fruit,stem and leaf	morphological characteristics and ITS	
20	*Colletotrichum aenigma*	*Tamarindus indica L*	fruit,stem and leaf	morphological characteristics and ITS	
21	*Colletotrichum brevisporum*	*Tamarindus indica L*	fruit,stem and leaf	morphological characteristics and ITS	
22	*Colletotrichum cobbittiense*	*Tamarindus indica L*	fruit,stem and leaf	morphological characteristics and ITS	
23	*Colletotrichum siamense*	*Tamarindus indica L*	fruit,stem and leaf	morphological characteristics and ITS	
24	*Colletotrichum fructicola*	*Tamarindus indica L*	fruit,stem and leaf	morphological characteristics and ITS	
25	*Diaporthe phaseolorum*	*Tamarindus indica L*	fruit,stem and leaf	morphological characteristics and ITS	
26	*Diaporthe arecae*	*Tamarindus indica L*	fruit,stem and leaf	morphological characteristics and ITS	
27	*Diaporthe ceratoxamiae*	*Tamarindus indica L*	fruit,stem and leaf	morphological characteristics and ITS	
28	*Diaporthe pseudomangzjirae*	*Tamarindus indica L*	fruit,stem and leaf	morphological characteristics and ITS	
29	*Diaporthe pseudooctlii*	*Tamarindus indica L*	fruit,stem and leaf	morphological characteristics and ITS	
30	*Diaporthe pseudophoenicicola*	*Tamarindus indica L*	fruit,stem and leaf	morphological characteristics and ITS	
31	*Aspergillus flavus*	*Tamarindus indica L*	fruit,stem and leaf	morphological characteristics and ITS	[10, 11]
32	*Aspergillus aculeatus*	*Tamarindus indica L*	fruit,stem and leaf	morphological characteristics and ITS	
33	*Aspergillus carbonarizls*	*Tamarindus indica L*	fruit,stem and leaf	morphological characteristics and ITS	
34	*Aspergillus tubingensis*	*Tamarindus indica L*	fruit,stem and leaf	morphological characteristics and ITS	
35	*Fusarium solani*	*Tamarindus indica L*	fruit,stem and leaf	morphological characteristics and ITS	
36	*Curvularial geniculatzrs*	*Tamarindus indica L*	fruit,stem and leaf	morphological characteristics and ITS	
37	*Curvularial lunata*	*Tamarindus indica L*	fruit,stem and leaf	morphological characteristics and ITS	
38	*Lasiodiplodia theobromae*	*Tamarindus indica L*	fruit,stem and leaf	morphological characteristics and ITS	
39	*Lasiodiplodia pseudotheobromae*	*Tamarindus indica L*	fruit,stem and leaf	morphological characteristics and ITS	
40	*Phyllosticta fallopiae*	*Tamarindus indica L*	fruit,stem and leaf	morphological characteristics and ITS	
41	*Nigrospora oryzae*	*Tamarindus indica L*	fruit,stem and leaf	morphological characteristics and ITS	
42	*Nigrospora lacticolonia*	*Tamarindus indica L*	fruit,stem and leaf	morphological characteristics and ITS	
43	*Penicillium rolfsii*	*Tamarindus indica L*	fruit,stem and leaf	morphological characteristics and ITS	

44	*Penicillium verruculosum*	*Tamarindus indica L*	fruit,stem and leaf	morphological characteristics and ITS	
45	*Trichoderma asperellum*	*Tamarindus indica L*	fruit,stem and leaf	morphological characteristics and ITS	
46	*Xylaria feejeensis*	*Tamarindus indica L*	fruit,stem and leaf	morphological characteristics and ITS	
47	*Sarcostroma bisetulatum*	*Tamarindus indica L*	fruit,stem and leaf	morphological characteristics and ITS	
48	*Botryosphaeria mamane*	*Tamarindus indica L*	fruit,stem and leaf	morphological characteristics and ITS	
49	*Truncospora tephropora*	*Tamarindus indica L*	fruit,stem and leaf	morphological characteristics and ITS	
50	*Ceratobasidium ramicola IBRLCM127*	*Curcuma mangga Valeton & Zijp*	rhizome	morphological characteristics and ITS	[6]
51	*Lasiodiplodia pseudotheobromae IBRL OS-64*	*Ocimum sanctum Linn*	leaf	morphological characteristics and ITS	[12]
52	*Colletotrichum sp. IBRL OS-27*	*Ocimum sanctum Linn*	leaf	morphological characteristics and ITS	
53	*Colletotrichum sp. IBRL OS-39*	*Ocimum sanctum Linn*	leaf	morphological characteristics and ITS	
54	*Lasiodiplodia sp. IBRL OS-64*	*Ocimum sanctum Linn*	leaf	morphological characteristics and ITS	
55	*Aspergillus sp. IBRL OS-65*	*Ocimum sanctum Linn*	leaf	morphological characteristics and ITS	[13]
56	*Aspergillus sp. IBRL OS-82*	*Ocimum sanctum Linn*	leaf	morphological characteristics and ITS	
57	*Muscodor sp. IBRL OS-94*	*Ocimum sanctum Linn*	leaf	morphological characteristics and ITS	
58	*Muscodor sp. IBRL OS-98*	*Ocimum sanctum Linn*	leaf	morphological characteristics and ITS	
59	*Lasiodiplodia pseudotheobromae IBRL OS-64*	*Ocimum sanctum Linn*	leaf	morphological characteristics and ITS	[14]
60	*Lasiodiplodia pseudotheobromae IBRL OS-64*	*Ocimum sanctum Linn*	leaf	morphological characteristics and ITS	[15]
61	*Muscodor sp. IBRL OS-94*	*Ocimum sanctum Linn*	leaf	morphological characteristics and ITS	[16]
62	*Penicillium minioluteum ED24*	*Orthosiphon stamineus Benth*	leaf	ITS	[17]
63	*Ceratobasidium ramicola IBRLCM127*	*Curcuma mangga Valeton & Zijp*	rhizome	morphological characteristics and ITS	[2]

64	*Aspergillus flavus IBRL-C8*	*Cassia siamea Lamk*	leaf	morphological characteristics and ITS	[18]
65	*Cytospora rhizophorae HAB16R12*	*Cinnamomum porrectum*	root	ITS	[19]
66	*Cytospora rhizophorae HAB16R13*	*Cinnamomum porrectum*	root	ITS	
67	*Cytospora rhizophorae HAB16R14*	*Cinnamomum porrectum*	root	ITS	
68	*Cytospora rhizophorae HAB16R18*	*Cinnamomum porrectum*	root	ITS	
69	*Cytospora rhizophorae HAB8R24*	*Cinnamomum porrectum*	root	ITS	
70	*Lasiodiplodia pseudotheobromae IBRL OS-64*	*Ocimum sanctum L.*	leaf	morphological characteristics and ITS	[20]
71	*Nigrospora sphaerica CL-OP30*	*Swietenia macrophylla*	leaf	morphological characteristics and ITS	[21]
72	*Diaporthe fraxini*	*Orthosiphon stamineus*	leaf	morphological characteristics and ITS	[22]
73	*Aspergillus sp. HAB10R12*	*Garcinia scortechinii*	leaf	morphological characteristics and ITS	[23]
74	*Colletotrichum gloeosporioides (strain OD3)*	*Oldenlandia diffusa*	leaf	morphological characteristics and ITS	[24]
75	*Fusarium fujikuroi*	*Dendrobium spp.*	root	ITS1/ITS4andITS5/ITS4	[25]
76	*Fusarium proliferatum*	*Dendrobium spp.*	root	ITS1/ITS4andITS5/ITS4	
77	*Fusarium oxysporum*	*Dendrobium spp.*	root	ITS1/ITS4andITS5/ITS4	
78	*Fusarium verticillioides*	*Dendrobium spp.*	root	ITS1/ITS4andITS5/ITS4	
79	*Trichoderma asperellum*	*Dendrobium spp.*	root	ITS1/ITS4andITS5/ITS4	
80	*Daldinia eschscholtzii*	*Dendrobium spp.*	root	ITS1/ITS4andITS5/ITS4	
81	*Nigrospora pyriformis*	*Dendrobium spp.*	root	ITS1/ITS4andITS5/ITS4	
82	*Buergenerula spartinae (isolate C28)*	*Cymbidium spp.*	root	ITS	
83	*Colletotrichum boninense (SM21)*	*Calamus castaneus*	spines	ITS,TEF-1α,β-Tubulin,GAPDH	[26]
84	*Colletotrichum fructicola (BP5)*	*Calamus castaneus*	spines	ITS,TEF-1α,β-Tubulin,GAPDH	
85	*Colletotrichum cliviae (SM25)*	*Calamus castaneus*	spines	ITS,TEF-1α,β-Tubulin,GAPDH	

86	*Diaporthe hongkongensis (SM42)*	*Calamus castaneus*	spines	ITS,TEF-1α,β-Tubulin,GAPDH	
87	*Diaporthe arengae (SM45)*	*Calamus castaneus*	spines	ITS,TEF-1α,β-Tubulin,GAPDH	
88	*Diaporthe cf. nobilis (BR67)*	*Calamus castaneus*	spines	ITS,TEF-1α,β-Tubulin,GAPDH	
89	*Neopestalotiopsis saprophytica (BP1)*	*Calamus castaneus*	spines	ITS,TEF-1α,β-Tubulin,GAPDH	
90	*Neopestalotiopsis formicarum (BP2)*	*Calamus castaneus*	spines	ITS,TEF-1α,β-Tubulin,GAPDH	
91	*Fusarium solani (BR92)*	*Calamus castaneus*	spines	ITS,TEF-1α,β-Tubulin,GAPDH	
92	*Fusarium oxysporum (BR86)*	*Calamus castaneus*	spines	ITS,TEF-1α,β-Tubulin,GAPDH	
93	*Lasiodiplodia pseudotheobromae IBRL OS-64*	*Ocimum sanctum*	spines	ITS,TEF-1α,β-Tubulin,GAPDH	[1]
94	*Diaporthe hongkongensis (SN1)*	*Gynura procumbens*	leaf	ITS	
95	*Phomopsis sp. (SN2)*	*Gynura procumbens*	leaf	ITS	
96	*Colletotrichum truncatum (SN3)*	*Gynura procumbens*	leaf	ITS	
97	*Mycoleptodiscus indicus (SN4)*	*Gynura procumbens*	leaf	ITS	
98	*Diaporthe longicolla (SN5)*	*Gynura procumbens*	leaf	ITS	
99	*Macrophomina phaseolina (SN6)*	*Gynura procumbens*	leaf	ITS	
100	*Beltraniella portoricensis (SN7)*	*Gynura procumbens*	leaf	ITS	[27]
101	*Colletotrichum asianum (SN8)*	*Gynura procumbens*	leaf	ITS	
102	*Colletotrichum brevisporum (SN9)*	*Gynura procumbens*	leaf	ITS	
103	*Fusarium incarnatum (SN10)*	*Gynura procumbens*	leaf	ITS	
104	*Colletotrichum gloeosporioides (SN11)*	*Gynura procumbens*	leaf	ITS	
105	*Pestalotiopsis sp. (SN12)*	*Gynura procumbens*	leaf	ITS	
106	*Diaporthe fraxini ED2*	*Orthosiphon stamineus*	leaf	ITS	[28]

107	*Lasiodiplodia pseudotheobromae IBRL OS-64*	*Ocimum sanctum*	leaf	ITS	[1]
108	*Muscodor sp. IBRL OS-94*	*Ocimum sanctum*	leaf	ITS	[29]
109	*Aspergillus austroafricanus MB1*	*Centella asiatica*	leaf, root, petioles, and stolon	ITS5,ITS4	[30]
110	*Colletotrichum karstii MM2*	*Centella asiatica*	leaf, root, petioles, and stolon	ITS5,ITS4	
111	*Fusarium solani 1 MM3*<br>*Fusarium falciforme MM4*	*Centella asiatica*	leaf, root, petioles, and stolon	ITS5,ITS4	
112	*Eutypella sp. MM5*	*Centella asiatica*	leaf, root, petioles, and stolon	ITS5,ITS4	
113	*Colletotrichum siamense MM9*	*Centella asiatica*	leaf, root, petioles, and stolon	ITS5,ITS4	
114	*Peroneutypa scoparia MM10*	*Centella asiatica*	leaf, root, petioles, and stolon	ITS5,ITS4	
115	*Phanerochaete stereoides MM12*	*Centella asiatica*	leaf, root, petioles, and stolon	ITS5,ITS4	
116	*Aspergillus oryzae MM13*	*Centella asiatica*	leaf, root, petioles, and stolon	ITS5,ITS4	
117	*Colletotrichum gigasporum MM14*	*Centella asiatica*	leaf, root, petioles, and stolon	ITS5,ITS4	
118	*Penicillium capsulatum MM15*	*Centella asiatica*	leaf, root, petioles, and stolon	ITS5,ITS4	
119	*Talaromyces pinophilus MM16*	*Centella asiatica*	leaf, root, petioles, and stolon	ITS5,ITS4	

120	*Fusarium solani 2 MM17*	*Centella asiatica*	leaf, root, petioles, and stolon	ITS5,ITS4	
121	*Colletotrichum tabaci 1MM18*	*Centella asiatica*	leaf, root, petioles, and stolon	ITS5,ITS4	
122	*Chaetomium globosum MM19*	*Centella asiatica*	leaf, root, petioles, and stolon	ITS5,ITS4	
123	*Fusarium striatum MM20*	*Centella asiatica*	leaf, root, petioles, and stolon	ITS5,ITS4	
124	*Perenniporia corticola MM21*	*Centella asiatica*	leaf, root, petioles, and stolon	ITS5,ITS4	
125	*Colletotrichum tabaci 2 MM23*	*Centella asiatica*	leaf, root, petioles, and stolon	ITS5,ITS4	
126	*Trichoderma reesei*	*Capsicum annuum L.*	fruit,stem and leaf	morphological characteristics and ITS	
127	*Hypoxylon sp.*	*Capsicum annuum L.*	leaf, fruit, stem	morphological characteristics and ITS	
128	*Aspergillus awamori*	*Capsicum annuum L.*	leaf, fruit, stem	morphological characteristics and ITS	
129	*Aulographum hederae*	*Capsicum annuum L.*	leaf, fruit, stem	morphological characteristics and ITS	[31]
130	*Bipolaris sorokiniana*	*Capsicum annuum L.*	leaf, fruit, stem	morphological characteristics and ITS	
131	*Patellaria atrata*	*Capsicum annuum L.*	leaf, fruit, stem	morphological characteristics and ITS	
132	*Aspergillus novofumigatus*	*Capsicum annuum L.*	leaf, fruit, stem	morphological characteristics and ITS	
133	*Aspergillus sp. HAB10R12*	*Garcinia scortechinii*	root	ITS	[32]
134	*Lasiodiplodia pseudotheobromae IBRL OS-64*	*Ocimum sanctum Linn.*	leaf	morphological characteristics and ITS	[33]
135	*Lasiodiplodia pseudotheobromae IBRL OS-64*	*Ocimum sanctum Linn.*	leaf	morphological characteristics and ITS	[34]
136	*Lasiodiplodia pseudotheobromae IBRL OS-64*	*Ocimum sanctum Linn.*	leaf	morphological characteristics and ITS	[15]

137	*Lasiodiplodia pseudotheobromae IBRL OS-64*	*Ocimum sanctum Linn.*	leaf	morphological characteristics and ITS	[12]
138	*Colletotrichum sp. (P1)*	*Pandanus sp., Alpinia sp.*	leaf	morphological characteristics	[35]
139	*Lentinus sp. (A1)*	*Pandanus sp., Alpinia sp.*	leaf	morphological characteristics	
140	*Zygomycota sp. (P2)*	*Pandanus sp., Alpinia sp.*	leaf	morphological characteristics	
141	*Nigrospora sphaerica*	*Catharanthus roseus*	leaf	morphological characteristics and ITS	[36]
142	*Macrophomina phaseolina*	*Catharanthus roseus*	leaf and root	morphological characteristics and ITS	
143	*Fusarium solani*	*Catharanthus roseus*	leaf and root	morphological characteristics and ITS	
144	*Colletotrichum gloeosporioides*	*Catharanthus roseus*	leaf and root	morphological characteristics and ITS	
145	*Ascomycota sp.*	*Melastoma malabathricum L.*	leaf, root and stems	morphological characteristics	[37]
146	*Zygomycota sp.*	*Melastoma malabathricum L.*	leaf, root and stems	morphological characteristics	
147	*Pestalotiopsis*	*Rhizophora mucronata*	leaf and bark	ITS	[38]
148	*Alternaria*	*Rhizophora mucronata*	leaf and bark	ITS	
149	*Cladosporium*	*Rhizophora mucronata*	leaf and bark	ITS	
150	*Fusarium lateritium*	*Rhizophora mucronata*	leaf and bark	ITS	
151	*Nigrospora oryzae*	*Rhizophora mucronata*	leaf and bark	ITS	
152	*Phoma sp.*	*Rhizophora mucronata*	leaf and bark	ITS	
153	*Xylaria sp.*	*Rhizophora mucronata*	leaf and bark	ITS	
154	*Lasiodiplodia pseudotheobromae IBRL OS-64*	*Ocimum sanctum Linn*	leaf	morphological characteristics and ITS	[39]
155	*Aspergillus sp. IBRL MP15 CCL*	*Swietenia macrophylla*	leaf	morphological characteristics and ITS	[40]
156	*Nigrospora sphaerica*	*Catharanthus roseus*	root and leaf	morphological characteristics, ITS1F and ITS4	[41]
157	*Sordariomycetes sp. (PDA)BL3 and (PDA)BL5*	*Strobilanthes crispus*	leaf	18S rDNA identification	[42]

158	*Penicillium purpurogenum ED76*	*Swietenia macrophylla*	leaf	ITS	[43]
159	*Colletotrichum sp.*	*Catharanthus roseus*	root and leaf	morphological characteristics, ITS1F and ITS4	[7]
160	*Macrophomina phaseolina*	*Catharanthus roseus*	root and leaf	morphological characteristics and ITS	
161	*Nigrospora sphaerica*	*Catharanthus roseus*	root and leaf	morphological characteristics and ITS	
162	*Fusarium solani*	*Catharanthus roseus*	root and leaf	morphological characteristics and ITS	
163	*Nigrospora sphaerica CL-OP30*	*Swietenia macrophylla*	leaf	ITS	[44]
164	*Aspergillus niger*	*Psilotum nudum*	whole plant	morphological characteristics	[45]
165	*Aspergillus flavus*	*Psilotum nudum*	whole plant	morphological characteristics	
166	*Aspergillus terreus*	*Psilotum nudum*	whole plant	morphological characteristics	
167	*Bipolaris sp.*	*Psilotum nudum*	whole plant	morphological characteristics	
168	*Coccidioides immitis*	*Psilotum nudum*	whole plant	morphological characteristics	
169	*Paracoccidioides brasiliensis*	*Psilotum nudum*	whole plant	morphological characteristics	
170	*Verticillium sp.*	*Psilotum nudum*	whole plant	morphological characteristics	
171	*Scedosporium apiospermum*	*Psilotum nudum*	whole plant	morphological characteristics	
172	*Nigrospora sphaerica CL-CP30*	*Swietenia macrophylla King*	leaf	ITS	[46]
173	*Fusarium fujikuroi*	*Cymbidium sp.*	root	ITS1/ITS4andITS5/ITS4	[47]
174	*Fusarium incarnatum*	*Cymbidium sp.*	root	ITS1/ITS4andITS5/ITS4	
175	*Fusarium proliferatum*	*Cymbidium sp.*	root	ITS1/ITS4andITS5/ITS4	
176	*Fusarium oxysporum*	*Cymbidium sp.*	root	ITS1/ITS4andITS5/ITS4	
177	*Lasiodiplodia theobromae*	*Cymbidium sp.*	root	ITS1/ITS4andITS5/ITS4	
178	*Nigrospora oryzae*	*Cymbidium sp.*	root	ITS1/ITS4andITS5/ITS4	
179	*Buergenerula spartinae*	*Cymbidium sp.*	root	ITS1/ITS4andITS5/ITS4	
180	*Colletotrichum siamense F272*	*Pereskia bleo*	leaf and stem	morphological characteristics, ITS1 and ITS4	[48]

181	*Colletotrichum sp. NK29*	*Pereskia bleo*	leaf and stem	morphological characteristics, ITS1 and ITS4
182	*Colletotrichum gloeosporioides*	*Pereskia bleo*	leaf and stem	morphological characteristics, ITS1 and ITS4
183	*Fusarium proliferatum*	*Pereskia bleo*	leaf and stem	morphological characteristics, ITS1 and ITS4
184	*Fusarium verticillioides strain jb111*	*Pereskia bleo*	leaf and stem	morphological characteristics, ITS1 and ITS4
185	*Penicillium simplicissimum strain KUC 5153*	*Pereskia bleo*	leaf and stem	morphological characteristics, ITS1 and ITS4
186	*Dothiodeomycetes sp. P15E6*	*Pereskia bleo*	leaf and stem	morphological characteristics, ITS1 and ITS4
187	*Fusarium oxysporum*	*Murraya koenigii*	leaf and stem	morphological characteristics, ITS1 and ITS4
188	*Fusarium verticillioides strain jb111*	*Oldenlandia diffusa*	leaf and stem	morphological characteristics, ITS1 and ITS4
189	*Ascomycota sp. AR-2010*	*Oldenlandia diffusa*	leaf and stem	morphological characteristics, ITS1 and ITS4
190	*Colletotrichum sp. GM414*	*Oldenlandia diffusa*	leaf and stem	morphological characteristics, ITS1 and ITS4
191	*Ascomycota sp.*	*Oldenlandia diffusa*	leaf and stem	morphological characteristics, ITS1 and ITS4
192	*Fusarium proliferatum*	*Cymbopogon citratus*	leaf and stem	morphological characteristics, ITS1 and ITS4
193	*Phoma sp.*	*Cymbopogon citratus*	leaf and stem	morphological characteristics, ITS1 and ITS4

194	*Colletotrichum sp. NK29*	*Cymbopogon citratus*	leaf and stem	morphological characteristics, ITS1 and ITS4	
195	*Dothideomycetes sp. P15E6*	*Cymbopogon citratus*	leaf and stem	morphological characteristics, ITS1 and ITS4	
196	*Colletotrichum kartsii*	*Centella asiatica*	stolon, leaf, root, petiole	ITS5 and ITS4	[49]
197	*Colletotrichum tabaci*	*Centella asiatica*	stolon, leaf, root, petiole	ITS5 and ITS4	
198	*Colletotrichum gigasporum*	*Centella asiatica*	stolon, leaf, root, petiole	ITS5 and ITS4	
199	*Colletotrichum siamense*	*Centella asiatica*	stolon, leaf, root, petiole	ITS5 and ITS4	
200	*Fusarium solani*	*Centella asiatica*	stolon, leaf, root, petiole	ITS5 and ITS4	
201	*Fusarium striatum*	*Centella asiatica*	stolon, leaf, root, petiole	ITS5 and ITS4	
202	*Fusarium sp.*	*Centella asiatica*	stolon, leaf, root, petiole	ITS5 and ITS4	
203	*Chaetomium globosum*	*Centella asiatica*	stolon, leaf, root, petiole	ITS5 and ITS4	
204	*Phomopsis asparagi*	*Centella asiatica*	stolon, leaf, root, petiole	ITS5 and ITS4	
205	*Phialemoniopsis sp.*	*Centella asiatica*	stolon, leaf, root, petiole	ITS5 and ITS4	
206	*Eutypella sp.*	*Centella asiatica*	stolon, leaf, root, petiole	ITS5 and ITS4	
207	*Peroneutypa scoparia*	*Centella asiatica*	stolon, leaf, root, petiole	ITS5 and ITS4	
208	*Ceratobasidium sp.*	*Centella asiatica*	stolon, leaf, root, petiole	ITS5 and ITS4	
209	*Perenniporia scoparia*	*Centella asiatica*	stolon, leaf, root, petiole	ITS5 and ITS4	
210	*Phanerochaete stereodes*	*Centella asiatica*	stolon, leaf, root, petiole	ITS5 and ITS4	
211	*Earliella scabrosa*	*Centella asiatica*	stolon, leaf, root, petiole	ITS5 and ITS4	
212	*Phyllosticta capitalensis*	*Centella asiatica*	stolon, leaf, root, petiole	ITS5 and ITS4	
213	*Aspergillus oryzae*	*Centella asiatica*	stolon, leaf, root, petiole	ITS5 and ITS4	
214	*Penicillium capsulatum*	*Centella asiatica*	stolon, leaf, root, petiole	ITS5 and ITS4	
215	*Talaromyces sp.*	*Centella asiatica*	stolon, leaf, root, petiole	ITS5 and ITS4	

Supplementary Table S2. Natural Products Derived from Malaysian Medicinal Plants from 1 Jan 2015 to 13 Mar 2025

No.	Types	Secondary metabolites	Fermentation	Extraction methods	Structural characterization	Fungal species	Medicinal Plant Host	Reference
1	Aromatic Aldehyde	benzaldehyde	Yeast extract sucrose (YES) broth	Column chromatography; MeOH/EtOAc elution	Gas chromatography–mass spectrometry (GC–MS)	*Lasiodiplodia pseudotheobromae* IBRL OS-64	*Ocimum sanctum*	[1]
2	Aromatic Alcohol	2-phenylethanol				*Lasiodiplodia pseudotheobromae* IBRL OS-64	*Ocimum sanctum*	
3	Fatty Alcohol	1-octanol				*Lasiodiplodia pseudotheobromae* IBRL OS-64	*Ocimum sanctum*	
4	Aromatic Aldehyde	2-phenylacetaldehyde.				*Lasiodiplodia pseudotheobromae* IBRL OS-64	*Ocimum sanctum*	
5	Enzyme	L-asparaginase	Potato Dextrose Agar (PDA) supplemented with L-asparagine (10 g/L) (Merck) and 0.3 mL of 2.5% phenol red dye.	Centrifugation at 13000 rpm and the supernatant was collected (crude enzyme)	Quantitative Nessler's test.	*Fusarium proliferatum* （isolate CCH）	*Cymbopogon citratus*	[3]
6	Flavonoids	Koburaside	Yeast extract sucrose broth	Dichloromethane	UPLC-QTOF-MS/MS	*Phyllosticta fallopiae* L67	*Aloe vera*	[4]
7	Flavonoids	Meliaedanoside A	Yeast extract sucrose broth	Dichloromethane	UPLC-QTOF-MS/MS	*Phyllosticta fallopiae* L67	*Aloe vera*	
8	Flavonoids	5,7,4'-Trihydroxy-8,3'-diprenylflavanone	Yeast extract sucrose broth	Dichloromethane	UPLC-QTOF-MS/MS	*Phyllosticta fallopiae* L67	*Aloe vera*	
9	Flavonoids	Kuwanon A	Yeast extract sucrose broth	Dichloromethane	UPLC-QTOF-MS/MS	*Phyllosticta fallopiae* L67	*Aloe vera*	
10	Flavonoids	3',4'-Dimethoxy-isoflavan-7,2'-di-O-β-D-glucoside	Yeast extract sucrose broth	Dichloromethane	UPLC-QTOF-MS/MS	*Phyllosticta fallopiae* L67	*Aloe vera*	
11	Chalcone derivative	Flavokawin A	Yeast extract sucrose broth	Dichloromethane	UPLC-QTOF-MS/MS	*Phyllosticta fallopiae* L67	*Aloe vera*	
12	Flavonoids	Ophiopogonanone B	Yeast extract sucrose broth	Dichloromethane	UPLC-QTOF-MS/MS	*Phyllosticta fallopiae* L67	*Aloe vera*	

13	Stilbene derivative	4'-Methylpinosylvin	Yeast extract sucrose broth	Dichloromethane	UPLC-QTOF-MS/MS	*Phyllosticta fallopiae* L67	*Aloe vera*	
14	Sesquiterpene lactone	Dendrosinene B	Yeast extract sucrose broth	Dichloromethane	UPLC-QTOF-MS/MS	*Phyllosticta fallopiae* L67	*Aloe vera*	
15	Biphenolic compound	Obovatol	Yeast extract sucrose broth	Dichloromethane	UPLC-QTOF-MS/MS	*Phyllosticta fallopiae* L67	*Aloe vera*	
16	Stilbene derivative	Moracin C	Yeast extract sucrose broth	Dichloromethane	UPLC-QTOF-MS/MS	*Phyllosticta fallopiae* L67	*Aloe vera*	
17	Bibenzyl derivative	4,4'-Dihydroxy-3,5-dimethoxybibenzyl	Yeast extract sucrose broth	Dichloromethane	UPLC-QTOF-MS/MS	*Phyllosticta fallopiae* L67	*Aloe vera*	
18	Diarylheptanoid	6-Gingeridione	Yeast extract sucrose broth	Dichloromethane	UPLC-QTOF-MS/MS	*Phyllosticta fallopiae* L67	*Aloe vera*	
19	Flavonoids	(2S)-3',4'-Methylenedioxy-5,7-dimethoxyflavane	Yeast extract sucrose broth	Dichloromethane	UPLC-QTOF-MS/MS	*Phyllosticta fallopiae* L67	*Aloe vera*	
20	Flavonoids	Kushenol I	Yeast extract sucrose broth	Dichloromethane	UPLC-QTOF-MS/MS	*Phyllosticta fallopiae* L67	*Aloe vera*	
21	Chromone derivative	6,7-Dimethoxy-2-(2'-p-methoxyphenylethyl) chromone	Yeast extract sucrose broth	Dichloromethane	UPLC-QTOF-MS/MS	*Phyllosticta fallopiae* L67	*Aloe vera*	
22	Flavonoids	Kushenol M	Yeast extract sucrose broth	Dichloromethane	UPLC-QTOF-MS/MS	*Phyllosticta fallopiae* L67	*Aloe vera*	
23	Polyketide	**3-hydroxy-5-methoxyhex-5-ene-2,4-dione**	YES medium	Ethyl acetate	column chromatography, HPLC,NMR,HRESI, HMBC	*Diaporthe* sp. ED2	*Orthosiphon stamineus Benth*	[8]
24	Amino acid derivative	Kynurenic acid	Yeast extract-sucrose broth (YES) and YES supplemented with 5 mg/L ros marinic acid (RA)	Ethyl acetate	NMR	*Diaporthe fraxini*	*Orthosiphon stamineus (Misai Kucing)*	[22]
25	Phenolic acid	Caffeic acid				*Diaporthe fraxini*	*Orthosiphon stamineus (Misai Kucing)*	
26	Phenolic acid	3,4-Dihydroxybenzeneacetic acid (a.k.a. Homogentisic acid)				*Diaporthe fraxini*	*Orthosiphon stamineus (Misai Kucing)*	
27	Phenolic acid	Gallic acid				*Diaporthe fraxini*	*Orthosiphon stamineus (Misai Kucing)*	

28	Nucleotide derivative	Deoxycytidine monophosphate				*Diaporthe fraxini*	*Orthosiphon stamineus (Misai Kucing)*	
29	Phenolic acid	Chlorogenic acid				*Diaporthe fraxini*	*Orthosiphon stamineus (Misai Kucing)*	
30	Aromatic amine / Tryptophan derivative	3-Hydroxyanthranilic acid				*Diaporthe fraxini*	*Orthosiphon stamineus (Misai Kucing)*	
31	Aromatic amine / Vitamin B complex intermediate	*p*-Aminobenzoic acid				*Diaporthe fraxini*	*Orthosiphon stamineus (Misai Kucing)*	
32	Phenylpropanoid	Cinnamic acid				*Diaporthe fraxini*	*Orthosiphon stamineus (Misai Kucing)*	
33	Aromatic acid derivative	Phenylglyoxylic acid				*Diaporthe fraxini*	*Orthosiphon stamineus (Misai Kucing)*	
34	Pyrimidine derivative	Orotic acid				*Diaporthe fraxini*	*Orthosiphon stamineus (Misai Kucing)*	
35	Indoleamine (Tryptophan derivative / Hormone)	Melatonin				*Diaporthe fraxini*	*Orthosiphon stamineus (Misai Kucing)*	
36	Alkaloids	**Aspergillinine A**	Potato dextrose agar(PDA)	Ethyl acetate	Sephadex® LH-20,semi-preparative HPLC,preparative HPLC,IR,UV,NMR, HMBC,NOESY,ECD ,HR-ESI-MS	*Aspergillus* sp. HAB10R12	*Garcinia scortechinii*	[23]
37	Alkaloids	**Aspergillinine B**				*Aspergillus* sp. HAB10R12	*Garcinia scortechinii*	
38	Alkaloids	**Aspergillinine C**				*Aspergillus* sp. HAB10R12	*Garcinia scortechinii*	

39	Alkaloids	**Aspergillinine D**				*Aspergillus* sp. HAB10R12	*Garcinia scortechinii*	
40	Diterpene pyrone	Aspergillipyrone A				*Aspergillus* sp. HAB10R12	*Garcinia scortechinii*	
41	Diterpene pyrone	Aspergillipyrone B				*Aspergillus* sp. HAB10R12	*Garcinia scortechinii*	
42	Diterpene pyrone	Aspergillipyrone C				*Aspergillus* sp. HAB10R12	*Garcinia scortechinii*	
43	Diterpene pyrone	Aspergillipyrone D				*Aspergillus* sp. HAB10R12	*Garcinia scortechinii*	
44	Benzamide derivative	Pranlukast	Potato dextrose broth (PDB)	Ethyl acetate	Thin layer chromatography (TLC),column chromatograph,LC-MS	*Buergenerula spartinae* (isolate C28)	*Cymbidium spp.*	[47]
45	Aromatic isocyanate	4,4'-Methylenediphenyl diisocyanate				*Buergenerula spartinae* (isolate C28)	*Cymbidium spp.*	
46	Steroid derivative	2-Methoxy-estrone-3-glucuronide				*Buergenerula spartinae* (isolate C28)	*Cymbidium spp.*	
47	Glycoside	Nigrose (Sakebioside)				*Buergenerula spartinae* (isolate C28)	*Cymbidium spp.*	
48	Chlorophyll derivative	Pyropheophorbide a				*Buergenerula spartinae* (isolate C28)	*Cymbidium spp.*	
49	Quinolone derivative	3-Quinolinecarboxylic acid, 7-amino-1-ethyl-6-fluoro-1,4-dihydro-4-oxo				*Buergenerula spartinae* (isolate C28)	*Cymbidium spp.*	
50	Alkaloids	Ergocornine				*Buergenerula spartinae* (isolate C28)	*Cymbidium spp.*	
51	Limonoid derivative	Epoxy (1,2α)-7-deacetoxy-7-oxo-deoxydihydrogedunin				*Buergenerula spartinae* (isolate C28)	*Cymbidium spp.*	
52	Heterocyclic compound	Debromopaoloxistatin				*Buergenerula spartinae* (isolate C28)	*Cymbidium spp.*	
53	Alkaloids	Ipecac (Psychotrine)				*Buergenerula spartinae* (isolate C28)	*Cymbidium spp.*	
54	Alkaloids	Rodiasine				*Buergenerula spartinae* (isolate C28)	*Cymbidium spp.*	
55	Fatty acid derivative	6,9-Heptadecadienoic acid				*Buergenerula spartinae* (isolate C28)	*Cymbidium spp.*	
56	Epoxy fatty acid	cis-9,10-Epoxystearic acid				*Buergenerula spartinae* (isolate C28)	*Cymbidium spp.*	

57	Steroid derivative	19-Norpregn-1,3,5(10)-trien-20-one-3,6,17-triol, (6b,17b)				*Buergenerula spartinae* (isolate C28)	*Cymbidium spp.*	
58	Aromatic sulfonic acid	N-Uncicelylbenzenesulfonic acid				*Buergenerula spartinae* (isolate C28)	*Cymbidium spp.*	
59	Steroid hormone derivative	2-Hydroxyestradiol				*Buergenerula spartinae* (isolate C28)	*Cymbidium spp.*	
60	Bile acid derivative	(25S)-3α,7α,12α-trihydroxy-5β-cholestan-26-oic acid				*Buergenerula spartinae* (isolate C28)	*Cymbidium spp.*	
61	Sulfonamide derivative	E3040				*Buergenerula spartinae* (isolate C28)	*Cymbidium spp.*	
62	Flavonoid derivative	4-O-Methylvaldosine				*Buergenerula spartinae* (isolate C28)	*Cymbidium spp.*	
63	Azole antifungal	Beconiazole				*Buergenerula spartinae* (isolate C28)	*Cymbidium spp.*	
64	Alkaloids	(−)-Osmosanine				*Buergenerula spartinae* (isolate C28)	*Cymbidium spp.*	
65	Phenylpropanoid derivative	Isoeugenol	Potato dextrose broth (PDB)	Ethyl acetate	GC–MS	*Diaporthe hongkongensis* (SN1) *Phomopsis* sp. (SN2) *Colletotrichum truncatum* (SN3) *Mycoleptodiscus indicus* (SN4) *Diaporthe longicolla* (SN5) *Macrophomina phaseolina* (SN6) *Beltraniella portoricensis* (SN7) *Colletotrichum asianum* (SN8) *Colletotrichum brevisporum* (SN9) *Fusarium incarnatum* (SN10) *Colletotrichum gloeosporioides* (SN11)	*Gynura procumbens*	[27]
66	Monoterpene alcohol	Terpinen-4-ol					*Gynura procumbens*	
67	Monoterpene oxide	Eucalyptol					*Gynura procumbens*	
68	Fatty acid	Oleic acid					*Gynura procumbens*	
69	Monoterpene hydrocarbon	β-Pinene					*Gynura procumbens*	
70	Monoterpene hydrocarbon	γ-Terpinene					*Gynura procumbens*	
71	Monoterpene	4-Carene					*Gynura procumbens*	

	hydrocarbon					*Pestalotiopsis* sp. (SN12)		
72	β2-Adrenoceptor agonist (synthetic compound)	Albuterol					*Gynura procumbens*	
73	Fatty acid	Octadecanoic acid (Stearic acid)					*Gynura procumbens*	
74	Sesquiterpene hydrocarbon	Caryophyllene					*Gynura procumbens*	
75	Sesquiterpene hydrocarbon	Aromadendrene					*Gynura procumbens*	
76	Sesquiterpene alcohol	Globulol					*Gynura procumbens*	
77	Flavonoid derivative	Hexamethylquercetagetin	Yeast extract sucrose broth , yeast extract sucrose broth supplemented with 5 mg/L rosmarinic acid.	Ethyl acetate	LC-HRMS	*Diaporthe fraxini* ED2	*Orthosiphon stamineus (Java tea)*	[28]
78	Quinoline derivative	Thioquinolactobactin				*Diaporthe fraxini* ED2	*Orthosiphon stamineus (Java tea)*	
79	Pyranone derivative	3-Acetyl-4-hydroxy-6-methyl-2H-pyran-2-one				*Diaporthe fraxini* ED2	*Orthosiphon stamineus (Java tea)*	
80	Alkaloids	N-Methyl-14-O-demethyllepi porphyrioxine				*Diaporthe fraxini* ED2	*Orthosiphon stamineus (Java tea)*	
81	Pyrone derivative	Vermopyrone				*Diaporthe fraxini* ED2	*Orthosiphon stamineus (Java tea)*	
82	Phenolic acid derivative	2-Amino-3-(3,4-dihydroxyphenyl)propanoic acid				*Diaporthe fraxini* ED2	*Orthosiphon stamineus (Java tea)*	

83	Bicyclic amino acid derivative	α-Amino-5-oxo-7-oxabicyclo[4.1.0]heptane-2-propanoic acid				*Diaporthe fraxini* ED2	*Orthosiphon stamineus (Java tea)*	
84	Coumarin derivative	12-Decarboxy-4′,5′-dihydromuscacaurin I				*Diaporthe fraxini* ED2	*Orthosiphon stamineus (Java tea)*	
85	Aromatic aldehyde	5-Acetyl-2-hydroxybenzaldehyde				*Diaporthe fraxini* ED2	*Orthosiphon stamineus (Java tea)*	
86	Alkaloids	Aculeatin A				*Diaporthe fraxini* ED2	*Orthosiphon stamineus (Java tea)*	
87	Alkaloids	Toxicol B				*Diaporthe fraxini* ED2	*Orthosiphon stamineus (Java tea)*	
88	Anthraquinone derivative	3-O-Demethylde hydroamorphigenin				*Diaporthe fraxini* ED2	*Orthosiphon stamineus (Java tea)*	
89	Sesquiterpenoid	**Asperginol A**	PDB	Acetone	IR,HPLC,NMR,HRESIMS	*Aspergillus* sp. HAB10R12	*Garcinia scortechinii*	[32]
90	Sesquiterpenoid	**Asperginol B**				*Aspergillus* sp. HAB10R12	*Garcinia scortechinii*	
91	Sesquiterpenoid	Asperginol C				*Aspergillus* sp. HAB10R12	*Garcinia scortechinii*	
92	Sesquiterpenoid	Asperginol D				*Aspergillus* sp. HAB10R12	*Garcinia scortechinii*	
93	Sesquiterpenoid	Asperginol E				*Aspergillus* sp. HAB10R12	*Garcinia scortechinii*	
94	Sesquiterpenoid	Asperginol F				*Aspergillus* sp. HAB10R12	*Garcinia scortechinii*	
95	Alkaloids	vinblastine	Yeast extract sucrose broth (YESB)	Dichloromethane	LC-MS	*Nigrospora sphaerica*	*Catharanthus roseus*	[41]
96	Alkaloids	Pyrrolo[1,2-a]pyrazine-1,4-dione	Potato dextrose agar(PDA)	Dichloromethane	GC–MS	Sordariomycetes sp.BL3 and Sordariomycetes sp.BL5	*Strobilanthes crispus*	[42]
97	Alcohols	1-Dodecanol				Sordariomycetes sp.BL3	*Strobilanthes crispus*	

98	Alcohols	n-Pentadecanol	Sordariomycetes sp.BL3	*Strobilanthes crispus*
99	Aldehydes	2-Heptenal, (Z)	Sordariomycetes sp.BL3	*Strobilanthes crispus*
100	Aldehydes	Nonanal	Sordariomycetes sp.BL3	*Strobilanthes crispus*
101	Aldehydes	2-Nonenal, (E)	Sordariomycetes sp.BL3	*Strobilanthes crispus*
102	Aldehydes	2-Decenal, (E)	Sordariomycetes sp.BL3	*Strobilanthes crispus*
103	Aldehydes	2,4-Dodecadienal, (E,E)	Sordariomycetes sp.BL3	*Strobilanthes crispus*
104	Aldehydes	1,8-Naphthalenedione, 8a-ethylperhydro	Sordariomycetes sp.BL3	*Strobilanthes crispus*
105	Aldehydes	3,5-di-tert-Butyl-4-hydroxybenzaldehyde	Sordariomycetes sp.BL3	*Strobilanthes crispus*
106	Aldehydes	Tetradecanal	Sordariomycetes sp.BL3	*Strobilanthes crispus*
107	Aldehydes	Dibutyl phthalate	Sordariomycetes sp.BL3 and Sordariomycetes sp.BL5	*Strobilanthes crispus*
108	Aldehydes	cis-9-Hexadecenal	Sordariomycetes sp.BL3	*Strobilanthes crispus*
109	Aldehydes	Ergotaman-3’,6’,18-trione, 9,10-dihydro-12’-hydroxy-2’-methyl-5’-(phenylmethyl)-,(5’.alpha,10.alpha)	Sordariomycetes sp.BL3	*Strobilanthes crispus*
110	Alkaloids	Decane, 3,7-dimethyl	Sordariomycetes sp.BL3	*Strobilanthes crispus*
111	Alkaloids	Undecane	Sordariomycetes sp.BL3 and Sordariomycetes sp.BL5	*Strobilanthes crispus*
112	Alkaloids	Nonane, 5-methyl-5-propyl	Sordariomycetes sp.BL3	*Strobilanthes crispus*
113	Alkaloids	Dodecane, 4,6-dimethyl	Sordariomycetes sp.BL3 and	*Strobilanthes crispus*

			Sordariomycetes sp.BL5	
114	Alkaloids	Eicosane	Sordariomycetes sp.BL3 and Sordariomycetes sp.BL5	*Strobilanthes crispus*
115	Alkaloids	Pentadecane	Sordariomycetes sp.BL3	*Strobilanthes crispus*
116	Alkaloids	2,6-Dimethyltridecanenitrile	Sordariomycetes sp.BL3	*Strobilanthes crispus*
117	Alkaloids	Decane, 1-iodo	Sordariomycetes sp.BL3	*Strobilanthes crispus*
118	Alkaloids	Tetracontane	Sordariomycetes sp.BL3	*Strobilanthes crispus*
119	Alkaloids	Tetrapentacontane	Sordariomycetes sp.BL3	*Strobilanthes crispus*
120	Alkaloids	5,10-Diethoxy-2,3,7,8-tetrahydro-1H,6H-dipyrrolo[1,2-a;1’,2’-d]pyrazine	Sordariomycetes sp.BL3	*Strobilanthes crispus*
121	Amino acid derivatives	DL-Alanyl-L-leucine	Sordariomycetes sp.BL3	*Strobilanthes crispus*
122	Others	Benzene, 1-[2-(2-chloroethoxy)ethoxy]-4-(1,1,3,3-tetramethylbutyl)	Sordariomycetes sp.BL3	*Strobilanthes crispus*
123	Others	3,4-Xylyl 3,5-di-tert-butylbenzoate	Sordariomycetes sp.BL3	*Strobilanthes crispus*
124	Carboxylic acid esters	Pentanoic acid, 3-hydroxy-ethyl ester	Sordariomycetes sp.BL3	*Strobilanthes crispus*
125	Carboxylic acid esters	1-(4-Hydroxy-3-methoxyphenyl)-1-ethoxyacetic acid ethyl ester, O-trimethylsilyl	Sordariomycetes sp.BL3	*Strobilanthes crispus*
126	Carboxylic acid esters	Tridecanoic acid, 3-hydroxy-, ethyl ester	Sordariomycetes sp.BL3	*Strobilanthes crispus*
127	Carboxylic acid esters	Hexadecanoic acid, methyl ester	Sordariomycetes sp.BL3 and	*Strobilanthes crispus*

			Sordariomycetes sp.BL5	
128	Carboxylic acid esters	Benzoic acid, 2,6-bis[(trimethylsilyl)oxy]-, trimethylsilyl ester	Sordariomycetes sp.BL3	*Strobilanthes crispus*
129	Carboxylic acid esters	9-Octadecenoic acid, methyl ester, (E)	Sordariomycetes sp.BL3 and Sordariomycetes sp.BL5	*Strobilanthes crispus*
130	Carboxylic acid esters	Octadecanoic acid, methyl ester	Sordariomycetes sp.BL3	*Strobilanthes crispus*
131	Carboxylic acid esters	12-Oxododecanoic acid, ethyl ester	Sordariomycetes sp.BL3	*Strobilanthes crispus*
132	Carboxylic acid esters	1,2-Benzenedicarboxylic acid, mono(2-ethylhexyl) ester	Sordariomycetes sp.BL3	*Strobilanthes crispus*
133	Carboxylic acids	Uric acid	Sordariomycetes sp.BL3 and Sordariomycetes sp.BL5	*Strobilanthes crispus*
134	Carboxylic acids	Octadecanoic acid	Sordariomycetes sp.BL3 and Sordariomycetes sp.BL5	*Strobilanthes crispus*
135	Others	Furan, 2-pentyl	Sordariomycetes sp.BL3	*Strobilanthes crispus*
136	Others	4-Piperidinone, 2,2,6,6-tetramethyl	Sordariomycetes sp.BL3	*Strobilanthes crispus*
137	Others	2-[di(tert-Butyl)silyloxymethyl] tetrahydrofuran	Sordariomycetes sp.BL3	*Strobilanthes crispus*
138	Others	2,5-di-O-Acetyl-3,4,6-tri-O-methyl-D-gluconitrile	Sordariomycetes sp.BL3	*Strobilanthes crispus*
139	Others	1-Nonadecene	Sordariomycetes sp.BL3	*Strobilanthes crispus*
140	Others	2H-Pyrrol-2-one,1,5-dihydro-4-(1-pyrrolidinyl)	Sordariomycetes sp.BL3	*Strobilanthes crispus*
141	Others	4(3H)-Pyrimidinone, 3-ethyl-2,6-dimethyl	Sordariomycetes sp.BL3	*Strobilanthes crispus*

142	Phenols	Phenol, m-tert-butyl	Sordariomycetes sp.BL3	*Strobilanthes crispus*
143	Phenols	Phenol, 2,4-bis(1,1-dimethylethyl)	Sordariomycetes sp.BL3 and Sordariomycetes sp.BL5	*Strobilanthes crispus*
144	Quinones	2,5-Cyclohexadiene-1,4-dione, 2,6-bis(1,1-dimethylethyl)	Sordariomycetes sp.BL3	*Strobilanthes crispus*
145	Quinones	2,5-Piperazinedione, 3-methyl-6-(1-methylethyl)	Sordariomycetes sp.BL3	*Strobilanthes crispus*
146	Quinones	3,6-Nonadecadione	Sordariomycetes sp.BL3	*Strobilanthes crispus*
147	Quinones	Pyrrolo[1,2-a]pyrazine-1,4-dione, hexahydro-3-(2-methylpropyl)	Sordariomycetes sp.BL3 and Sordariomycetes sp.BL5	*Strobilanthes crispus*
148	Quinones	3,6-Diisopropylpiperazin-2,5-dione	Sordariomycetes sp.BL3	*Strobilanthes crispus*
149	Quinones	Pyrrolo[1,2-a]pyrazine-1,4-dione, hexahydro-3-(phenylmethyl)	Sordariomycetes sp.BL3 and Sordariomycetes sp.BL5	*Strobilanthes crispus*
150	Siloxanes	Cyclotetrasiloxane, octamethyl	Sordariomycetes sp.BL3;Sordariomycetes sp.BL3	*Strobilanthes crispus*
151	Siloxanes	Silane, [3-(2,3-epoxypropoxy) propyl]ethoxydimethyl	Sordariomycetes sp.BL3	*Strobilanthes crispus*
152	Siloxanes	Cyclopentasiloxane, decamethyl	Sordariomycetes sp.BL3;Sordariomycetes sp.BL3	*Strobilanthes crispus*
153	Siloxanes	Silane, dimethyl(2-methoxyethoxy)hexyloxy	Sordariomycetes sp.BL3	*Strobilanthes crispus*
154	Siloxanes	Allyloxydi(tert-butyl)silane	Sordariomycetes sp.BL3	*Strobilanthes crispus*
155	Siloxanes	Silane, trimethyl(1-methylbutoxy)	Sordariomycetes sp.BL3	*Strobilanthes crispus*
156	Siloxanes	Cyclohexasiloxane, dodecamethyl	Sordariomycetes sp.BL3	*Strobilanthes crispus*

157	Siloxanes	Hexasiloxane, 1,1,3,3,5,5,7,7,9,9,11,11-dodecamethyl	Sordariomycetes sp.BL3	*Strobilanthes crispus*
158	Siloxanes	Cyclooctasiloxane, hexadecamethyl	Sordariomycetes sp.BL3 and Sordariomycetes sp.BL5	*Strobilanthes crispus*
159	Siloxanes	Cyclononasiloxane, octadecamethyl	Sordariomycetes sp.BL3 and Sordariomycetes sp.BL5	*Strobilanthes crispus*
160	Alcohols	2-Allyl-4-methylphenol	Sordariomycetes sp. BL5	*Strobilanthes crispus*
161	Alcohols	3,3,4-Trimethyl-4-(4-methylphenyl)cyclopentanol	Sordariomycetes sp. BL5	*Strobilanthes crispus*
162	Alcohols	Behenic alcohol	Sordariomycetes sp. BL5	*Strobilanthes crispus*
163	Aldehydes	Benzaldehyde, 2,4-dimethyl	Sordariomycetes sp. BL5	*Strobilanthes crispus*
164	Aromatic hydrocarbons	Benzene, 4-chloro-1,2-dimethyl	Sordariomycetes sp. BL5	*Strobilanthes crispus*
165	Aromatic hydrocarbons	1,3,5-Tris(trimethylsiloxy) benzene	Sordariomycetes sp. BL5	*Strobilanthes crispus*
166	Aromatic hydrocarbons	Benzene, 1-methyl-4-(1,2,2-trimethylcyclopentyl), (R)	Sordariomycetes sp. BL5	*Strobilanthes crispus*
167	Aromatic hydrocarbons	1,3-Benzenediol, o-ethoxycarbonyl	Sordariomycetes sp. BL5	*Strobilanthes crispus*
168	Carboxylic acid esters	1-(4-Hydroxy-3-methoxyphenyl)-1-ethoxyacetic acid ethyl ester	Sordariomycetes sp. BL5	*Strobilanthes crispus*
169	Carboxylic acid esters	Octadecanoic acid, 2-[(trimethylsilyl)oxy], methyl ester	Sordariomycetes sp. BL5	*Strobilanthes crispus*
170	Carboxylic acid esters	Tridecanoic acid, 4,8,12-trimethyl-, methyl ester	Sordariomycetes sp. BL5	*Strobilanthes crispus*

171	Carboxylic acid esters	Acetic acid, [bis[(trimethylsilyl)oxy]phosphinyl], trimethylsilyl ester	Sordariomycetes sp. BL5	*Strobilanthes crispus*
172	Carboxylic acid esters	Dodecanoic acid, methyl ester	Sordariomycetes sp. BL5	*Strobilanthes crispus*
173	Carboxylic acid esters	Benzoic acid, 2,6-bis[(trimethylsilyl)oxy]-, trimethylsilyl ester	Sordariomycetes sp. BL5	*Strobilanthes crispus*
174	Carboxylic acid esters	1,2-Benzenedicarboxylic acid, mono(2-ethylhexyl) ester	Sordariomycetes sp. BL5	*Strobilanthes crispus*
175	Alkaloids	5,10-Diethoxy-2,3,7,8-tetrahydro-1H,6H-dipyrrolo[1,2-a;1',2'-d]pyrazine	Sordariomycetes sp. BL5	*Strobilanthes crispus*
176	Ketones	3,6-Nonadecadione	Sordariomycetes sp. BL5	*Strobilanthes crispus*
177	Ketones	Piperazine-3,5-dione, 1-tetradecanoyl	Sordariomycetes sp. BL5	*Strobilanthes crispus*
178	Others	1-Butyl(dimethyl)siloxypropane	Sordariomycetes sp. BL5	*Strobilanthes crispus*
179	Others	2-Coumaranone	Sordariomycetes sp. BL5	*Strobilanthes crispus*
180	Others	(4-Hydroxy-3-methoxyphenyl) ethylene glycol tris(trimethylsilyl) ether	Sordariomycetes sp. BL5	*Strobilanthes crispus*
181	Others	Acetophenone, 4'-hydroxy	Sordariomycetes sp. BL5	*Strobilanthes crispus*
182	Others	Heptadecane	Sordariomycetes sp. BL5	*Strobilanthes crispus*
183	Others	4-Amino-7-diethylamino-chromen-2-one	Sordariomycetes sp. BL5	*Strobilanthes crispus*
184	Others	Tetrahydrofuran-2-one, 5-[1-hydroxyhexyl]	Sordariomycetes sp. BL5	*Strobilanthes crispus*
185	Others	4-Amino-5-imidazole carboxamide, N,N,O-tris(trimethylsilyl)	Sordariomycetes sp. BL5	*Strobilanthes crispus*
186	Others	Methyl tetradecanoate	Sordariomycetes sp. BL5	*Strobilanthes crispus*

187	Others	1,3-Dimethyl-3,4,5,6-tetrahydro-2(1H)-pyrimidinone				Sordariomycetes sp. BL5	*Strobilanthes crispus*	
188	Others	Cyclopentanone, 3,3,4-trimethyl-4-(4-methylphenyl)				Sordariomycetes sp. BL5	*Strobilanthes crispus*	
189	Others	1-Nonadecene				Sordariomycetes sp. BL5	*Strobilanthes crispus*	
190	Others	Isopropyl myristate				Sordariomycetes sp. BL5	*Strobilanthes crispus*	
191	Others	Eicasone				Sordariomycetes sp. BL5	*Strobilanthes crispus*	
192	Siloxanes	Trisiloxane, 1,1,1,5,5,5-hexamethyl-3,3-bis[(trimethylsilyl)oxy]				Sordariomycetes sp. BL5	*Strobilanthes crispus*	
193	Carbazoles	7-Methyl-7H-dibenzo[b,g]carbozole	YES medium	Acetone,dichloromethane	GC-MS	*Penicillium purpurogenum* ED76	*Swietenia macrophylla*	[43]
194	Siloxanes	Dodecamethylcyclohexasiloxane				*Penicillium purpurogenum* ED76	*Swietenia macrophylla*	
195	Siloxanes	Tetradecamethylcycloheptasiloxane				*Penicillium purpurogenum* ED76	*Swietenia macrophylla*	
196	Steroidals	Stigmastan-3,5,22-trien				*Penicillium purpurogenum* ED76	*Swietenia macrophylla*	
197	Steroidals	Stigmasterol				*Penicillium purpurogenum* ED76	*Swietenia macrophylla*	
198	Steroidals	Cyclocholest-22-en-6-one				*Penicillium purpurogenum* ED76	*Swietenia macrophylla*	

References

1. Mat Jalil, M.Ibrahim, D. Volatile Bioactive Compounds from Lasiodiplodia pseudotheobromae IBRL OS-64, an Endophytic Fungus Residing in the Leaf of Ocimum sanctum. *HAYATI Journal of Biosciences*. **2022**, 29, 570-585.
2. Muazzam, K.Ibrahim, D. Anti-MRSA activity of ethyl acetate crude extract from endophytic fungus Ceratobasidium ramicola IBRLCM127 isolated from rhizome of Curcuma mangga Valeton & Zijp. *IOP Conference Series: Earth and Environmental Science*. **2021**, 756, 012040.
3. Yap, L.S.; Lee, W.L.Ting, A.S.Y. Optimization of L-asparaginase production from endophytic Fusarium proliferatum using OFAT and RSM and its cytotoxic evaluation. *J Microbiol Methods*. **2021**, 191, 106358.
4. Taher, M.A.; Tan, W.N.; Chear, N.J.; Leong, C.R.; Rashid, S.A.Tong, W.Y. Metabolites characterisation of endophytic Phyllosticta fallopiae L67 isolated from Aloe vera with antimicrobial activity on diabetic wound microorganisms. *Nat Prod Res*. **2023**, 37, 1674-1679.
5. Zanudin, N.A.M.; Hasan, N.a.Mansor, P. Identification and characterization of endophytic fungi from Garcinia atroviridis for potential antagonistic against phytopathogenic, Colletotrichum gloeosporioides. *Malaysian Applied Biology*. **2023**, 52, 97-106.
6. Abdul Rahman, K.A.M.; Abdul Rahim, M.S.A.; Zarkasi, K.Z.Ibrahim, D. Enhancement of Anti-MRSA Potential Produced by an Endophytic Fungus Ceratobasidium Ramicola IBRLCM127 via Submerged Fermentation System. *Malaysian Journal of Medicine & Health Sciences*. **2023**, 19.
7. Ayob, F.W.Simarani, K. Endophytic filamentous fungi from a Catharanthus roseus: Identification and its hydrolytic enzymes. *Saudi Pharmaceutical Journal*. **2016**, 24, 273-278.
8. Yenn, T.W.; Ring, L.C.; Nee, T.W.; Khairuddean, M.; Zakaria, L.Ibrahim, D. Endophytic Diaporthe sp. ED2 produces a novel anti-candidal ketone derivative. *Journal of Microbiology and Biotechnology*. **2017**, 27, 1065-1070.
9. Taufiq, M.M.J.Darah, I. Effect of ethyl acetate crude extract of Lasiodiplodia pseudotheobromae IBRL OS-64 against oral cavity bacteria with emphasis on Streptococcus mutans. *Journal of Applied Pharmaceutical Science*. **2019**, 9, 078-085.
10. Mohd Zainudin, N.A.I.; Zaini, N.; Nizam, N.; Abidin, D.Nazri, N. Diversity of endophytic fungi associated with fruits and leaves of Tamarindus indica L. based on its ribosomal DNA sequences. *BIOTROPIA*. **2021**, 28, 221-230.
11. Zainudin, N.A.I.M.; Zaini, N.A.M.; Nizam, N.H.M.; Abidin, D.F.Z.A.Z.Nazri, N.I.A.M. Diversity of endophytic fungi associated with fruits and leaves of Tamarindus indica L. based on its ribosomal DNA sequences. *Biotropia*. **2021**, 28, 221-230.
12. Taufiq, M.M.J.Darah, I. Biological Activity of Lasiodiplodia pseudotheobromae IBRL OS- 64 Extracts, an Endophytic Fungus Isolated from Medicinal Herb, Ocimum sanctum Against Foodborne Diarrhea-Caused Bacteria. *Pharmacognosy Journal*. **2020**, 12.

13. Mat Jalil, M.; Yahya, M.F.Z.R.; Zakaria, N.A.Ibrahim, D. Antifungal activity of endophytic fungi associated with *Ocimum sanctum*. *Jurnal Teknologi*. **2024**, 86, 149-158.
14. Mat Jalil, M.Ibrahim, D. Anti-MRSA of the ethyl acetate crude extract from Lasiodiplodia pseudotheobromae IBRL OS-64, an endophytic fungus isolated from leaf of Ocimum sanctum Linn. *International Journal of Pharmacy and Pharmaceutical Sciences*. **2018**, 10, 50.
15. Mat Jalil, M.Ibrahim, D. Antibacterial and Antibiofilm Activities of Crude Extract of Lasiodiplodia pseudotheobromae IBRL OS-64 against Foodborne Bacterium, Yersinia enterocolitica. *Journal of Pharmaceutical Research International*. **2020**, 32, 87-102.
16. Ibrahim, M.T.M.J.D. Anti-yeast activity of endophytic fungi isolated from medicinal herb Ocimum sanctum with emphasis on Candida albicans. *Research Journal of Biotechnology*. **2022**, 17, 1-12.
17. Yenn, T.; Ang, S.N.; Zakaria, L.; Ibrahim, D.Leong, C.R. Anti-MRSA Activity of Penicillium minioluteum ED24. *Journal of Chemical and Pharmaceutical Sciences*. **2015**, 8, 646-650.
18. Darah Ibrahim, N., Lim Sheh Hong. Anti-candidal activity of Aspergillus flavus IBRL-C8, an endophytic fungus isolated from Cassia siamea Lamk leaf. *Journal of Applied Pharmaceutical Sciences*. **2018**, 8, 083-087.
19. Harun, A.; Vidyadaran, S.; Lim, S.M.; Cole, A.L.Ramasamy, K. Malaysian endophytic fungal extracts-induced anti-inflammation in Lipopolysaccharide-activated BV-2 microglia is associated with attenuation of NO production and, IL-6 and TNF-α expression. *BMC Complement Altern Med*. **2015**, 15, 166.
20. Jalil, M.T.M.; Zakaria, N.A.; Yahya, M.F.Z.R.; Mohamad, S.A.S.Ibrahim, D. Assessment of Biological Activity, Total Phenolic Content, and Cytotoxicity of Ethyl Acetate Extracts from an Endophytic Fungus, Lasiodiplodia pseudotheobromae IBRL OS-64. *HAYATI Journal of Biosciences*. **2025**, 32, 445-458.
21. Mokhtar, N.; Ibrahim, D.; Zulfakar, S.; Zakaria, L.Zarkasi, K. Antibiofilm activity of Nigrospora sphaerica CL-OP30 endophytic extract against Streptococcus mutans - the causative agent of dental caries. *Malaysian Journal of Microbiology*. **2023**.
22. Nagarajan, K.; Ibrahim, B.; Bawadikji, A.A.; Khaw, K.Y.; Tong, W.Y.; Leong, C.R.; Ramanathan, S.Tan, W.N. Characterization of Metabolites in an Endophytic Fungus Diaporthe fraxini via NMR-based Metabolomics and Cholinesterase Inhibitory Activity. *Applied Biochemistry and Microbiology*. **2023**, 59, 316-322.
23. Alkhdhairawi, A.; Loo, J.; Abd Mutalib, N.; Abd Latip, N.; Manshoor, N.; Bakar, H.; Nagojappa, N.Weber, J.-F. Diketopiperazine and isoindolinone alkaloids from the endophytic fungus Aspergillus sp. HAB10R12. *Phytochemistry*. **2023**, 211, 113685.
24. Yap, L.S.; Leng, L.W.and Ting, A.S.Y. Bioprocessing and purification of extracellular L-asparaginase produced by endophytic Colletotrichum gloeosporioides and its anticancer activity. *Preparative Biochemistry & Biotechnology*. **2023**, 53, 653-671.
25. Chua, R.W.; Song, K.P.Ting, A.S.Y. Comparative analysis of antimicrobial compounds from endophytic Buergenerula spartinae from orchid. *Antonie Van Leeuwenhoek*. **2023**, 116, 1057-1072.

26. Azuddin, N.F.; Mohd, M.H.; Nik Rosely, N.F.; Mansor, A.Zakaria, L. Evaluation of the pathogenicity of endophytic fungi isolated from spines of rattan (Calamus castaneus) against other plant hosts. *J Appl Microbiol*. **2022**, 133, 3228-3238.
27. Jamal, H.A.A.; Husaini, A.; Sing, N.N.; Roslan, H.A.; Zulkharnain, A.Akinkunmi, W.A. Characterization of bioactive compounds produced by endophytic fungi isolated from Gynura procumbens (Sambung Nyawa). *Braz J Microbiol*. **2022**, 53, 2343.
28. Tan, W.N.; Nagarajan, K.; Lim, V.; Azizi, J.; Khaw, K.Y.; Tong, W.Y.; Leong, C.R.Chear, N.J. Metabolomics Analysis and Antioxidant Potential of Endophytic Diaporthe fraxini ED2 Grown in Different Culture Media. *J Fungi (Basel)*. **2022**, 8.
29. Mat Jalil, M.Ibrahim, D. Muscodor sp. IBRL OS-94, A Promising Endophytic Fungus of Ocimum sanctum with Antimicrobial Activity. *Pharmaceutical Sciences*. **2021**, 27, 268-280.
30. Radiastuti, N.; Susilowati, D.N.; Nurhasni; Rustanti, L.P.Tambunan, I.R. Potential of endophytic fungi deriving from Asiatic pennyworth to produce antioxidants. *Indonesian Journal of Agricultural Science*. **2021**, 22, 58–65.
31. Zanudin, N.A.M.; Hasan, N.A.; Noruddin, N.F.N.; Ahmad Wakid, S.Hasbullah, N.I. Preliminary screening of endophytic fungi from capsicum annuum l. For biocontrol activity against colletotrichum gloeosporoioides. *Malaysian Journal of Biochemistry and Molecular Biology*. **2021**, 24, 175-184.
32. Al-Khdhairawi, A.A.Q.; Low, Y.Y.; Manshoor, N.; Arya, A.; Jelecki, M.; Alshawsh, M.A.; Kamran, S.; Suliman, R.S.; Low, A.; Shivanagere Nagojappa, N.B., et al. Asperginols A and B, Diterpene Pyrones, from an Aspergillus sp. and the Structure Revision of Previously Reported Analogues. *J Nat Prod*. **2020**, 83, 3564-3570.
33. Suhaimi, M.T.M.J.D.I.N.S.M. Time-kill study and morphological changes of Proteus mirabilis cells exposed to ethyl acetate crude extract of Lasiodiplodia pseudotheobromae IBRL OS-64. *Malaysian Journal of Microbiology*. **2020**, 16, pp. 219-228.
34. Ibrahim, M.T.M.J.D. Anti-candidal activity of ethyl acetate crude extract of endophytic fungus, Lasiodiplodia pseudotheobromae IBRL OS-64 against Candida albicans. *International Journal of Pharmaceutical Research*. **2020**, 12, 257-265.
35. Hasan, N.A.Zaini, M. Isolation and identification of Endophytic Fungi of Pandanus sp. and Alpinia sp. from Reserve Forest UiTM Negeri Sembilan. *Journal of Academia*. **2019**, 7, 124-129.
36. Ayob, F.W.Simarani, K. Antioxidants and Phytochemical Analysis of Endophytic Fungi Isolated from a Medicinal Plant Catharanthus roseus. *Borneo Journal of Sciences and Technology*. **2019**, 1, 62-68.
37. Hasan, N.A.Mohd Sudin, N.I.N. Diversity and Antifungal activity of endophytic fungi associated with Melastoma malabathricum L. *Journal of Applied Biological Sciences*. **2019**, 13, 154-159.
38. Hamzah, T.N.T.; Lee, S.Y.; Hidayat, A.; Terhem, R.; Faridah-Hanum, I.Mohamed, R. Diversity and Characterization of Endophytic Fungi Isolated From the Tropical Mangrove Species, Rhizophora mucronata, and Identification of Potential Antagonists Against the Soil-Borne Fungus, Fusarium solani. *Front Microbiol*. **2018**, 9, 1707.

39. Mohd Taufiq Mat Jalil, D.I. Effect of ethyl acetate crude extract of Lasiodiplodia pseudotheobromae IBRL OS-64 against oral cavity bacteria with emphasis on Streptococcus mutans. *International Journal of Pharmaceutical Sciences and Research*. **2019**, 9, 078–085.
40. Leong CheanRing, L.C.; Adib Arifah, B.M.; Syarifah Abdul Rashid, S.A.R.; Ang SweeNgim, A.S.; Tan WenNee, T.W.; Tong WoeiYenn, T.W.Ibrahim, D. Antimicrobial activity of Aspergillus sp. IBRL MP15 CCL, an endophytic fungus isolated from Swietenia macrophylla leaf against human pathogens. *Malaysian Journal of Microbiology*. **2018**, 14, 49–54.
41. Ayob, F.W.; Simarani, K.; Zainal Abidin, N.Mohamad, J. First report on a novel Nigrospora sphaerica isolated from Catharanthus roseus plant with anticarcinogenic properties. *Microb Biotechnol*. **2017**, 10, 926-932.
42. Jinfeng, E.C.; Mohamad Rafi, M.I.; Chai Hoon, K.; Kok Lian, H.Yoke Kqueen, C. Analysis of chemical constituents, antimicrobial and anticancer activities of dichloromethane extracts of Sordariomycetes sp. endophytic fungi isolated from Strobilanthes crispus. *World J Microbiol Biotechnol*. **2017**, 33, 5.
43. Yenn, T.; Ibrahim, D.; Lee, K.C.; Ab Rashid, S.; Leong, C.R.; Wen Nee, T.Noor, M. Antimicrobial efficacy of endophytic Penicillium purpurogenum ED76 against clinical pathogens and its possible mode of action. *Korean Journal of Microbiology*. **2017**, 53, 1-7.
44. Ibrahim, D.; Yenn, T.; Zakaria, L.Lim, S.-H. Effect of the Extract of Endophytic fungus, Nigrospora sphaerica CL-OP 30, Against the Growth of Methicillin- Resistant Staphylococcus aureus (MRSA) and Klebsiella pneumonia cells. *Tropical Journal of Pharmaceutical Research*. **2015**, 14, 2091.
45. Eliya, A.; Talip, N.Ibrahim, N. Anatomy of symbiotic fungal endophytes in psilotum nudum (L.) p. Beauv. *Malaysian Applied Biology*. **2015**, 44, 11-18.
46. Woei, I.C.; Ibrahim, D.; Daud, S.A.; Salikin, N.H.; Yenn, T.W.Ab Rashid, S. Antimicrobial activities of the ethyl acetate extract of endophytic Nigrospora sphaerica CL-CP30 against foodborne pathogens. *Malaysian Journal of Microbiology*. **2024**, 20.
47. Chua, R.W.; Song, K.P.Ting, A.S.Y. Antioxidant properties and L-asparaginase activities of endophytic fungi from Cymbidium orchids. *Folia Microbiol (Praha)*. **2024**, 69, 713-722.
48. Chow, Y.Ting, A.S.Y. Endophytic l-asparaginase-producing fungi from plants associated with anticancer properties. *Journal of Advanced Research*. **2015**, 6, 869-876.
49. Radiastuti, N.; Bahalwan, H.Susilowati, D. Phylogenetic study of endophytic fungi associated with Centella asiatica from Bengkulu and Malaysian accessions based on the ITS rDNA sequence. *Biodiversitas*. **2019**, 20, 1248-1258.
